# Single-cell and genetic multi-omics analysis combined with experiments confirmed the signature and potential targets of cuproptosis in hepatocellular carcinoma

**DOI:** 10.3389/fcell.2023.1240390

**Published:** 2023-09-08

**Authors:** Feng Cao, Yong Qi, Wenyong Wu, Xutong Li, Chuang Yang

**Affiliations:** ^1^ Department of General, Visceral and Transplantation Surgery, University Hospital RWTH Aachen, Aachen, Germany; ^2^ Department of General Surgery, The First Hospital of Anhui Medical University, Hefei, China; ^3^ Department of Infectious Diseases, The First Affiliated Hospital of Anhui Medical University, Hefei, China; ^4^ Department of Visceral, Transplant, Thoracic and Vascular Surgery, University Hospital of Leipzig, Leipzig, Germany

**Keywords:** hepatocellular carcinoma, cuproptosis, programmed cell death, immune microenvironment, single-cell RNA-sequencing, molecular docking

## Abstract

**Background:** Cuproptosis, as a recently discovered type of programmed cell death, occupies a very important role in hepatocellular carcinoma (HCC) and provides new methods for immunotherapy; however, the functions of cuproptosis in HCC are still unclear.

**Methods:** We first analyzed the transcriptome data and clinical information of 526 HCC patients using multiple algorithms in R language and extensively described the copy number variation, prognostic and immune infiltration characteristics of cuproptosis related genes (CRGs). Then, the hub CRG related genes associated with prognosis through LASSO and Cox regression analyses and constructed a prognostic prediction model including multiple molecular markers and clinicopathological parameters through training cohorts, then this model was verified by test cohorts. On the basis of the model, the clinicopathological indicators, immune infiltration and tumor microenvironment characteristics of HCC patients were further explored via bioinformation analysis. Then, We further explored the key gene biological function by single-cell analysis, cell viability and transwell experiments. Meantime, we also explored the molecular docking of the hub genes.

**Results:** We have screened 5 hub genes associated with HCC prognosis and constructed a prognosis prediction scoring model. And the model results showed that patients in the high-risk group had poor prognosis and the expression levels of multiple immune markers, including PD-L1, CD276 and CTLA4, were higher than those patients in the low-risk group. We found a significant correlation between risk score and M0 macrophages and memory CD4^+^ T cells. And the single-cell analysis and molecular experiments showed that BEX1 were higher expressed in HCC tissues and deletion inhibited the proliferation, invasion and migration and EMT pathway of HCC cells. Finally, it was observed that BEX1 could bind to sorafenib to form a stable conformation.

**Conclusion:** The study not only revealed the multiomics characteristics of CRGs in HCC but also constructed a new high-accuracy prognostic prediction model. Meanwhile, BEX1 were also identified as hub genes that can mediate the cuproptosis of hepatocytes as potential therapeutic targets for HCC.

## 1 Introduction

The latest global cancer statistics indicate that there are more than 900,000 new cases of liver cancer and 800,000 deaths per year, and its morbidity and mortality rank seventh and third among all cancers, respectively (Sung et al., 2021), which pose a serious threat to human health and safety (Llovet et al., 2021). Among them, nearly 90% of the cases are hepatocellular carcinoma (HCC) and it is also the most common type of pathology (Llovet et al., 2016). HCC progresses rapidly and stealthily, with a high degree of malignancy, the prognosis of HCC is very poor and the 5-year survival rate is below 20% (Craig et al., 2020). Although alpha-fetoprotein (AFP) is currently widely used in early HCC screening and prognosis assessment, it is affected not only by many non-hepatic carcinoma-related factors, but its expression level is also significantly increased in other diseases, such as acute viral hepatitis (AHA), resulting in low sensitivity and specificity (Huo et al., 2018; Cai et al., 2019). For lack of effective biomarkers, most patients are in the advanced stages when they are diagnosed with HCC. Although a variety of modalities including surgical resection, radiofrequency ablation, trans-arterial chemoembolization, systemic chemotherapy and liver transplantation have been significantly developed, the clinical effect is still very limited. In addition, the complex pathogenesis of HCC is also an important factor in its high mortality, which involves multiple molecular mechanisms, such as cell death regulation, genetic mutation, tumor heterogeneity, immune regulation, and dysregulation of the tumor microenvironment (Kurebayashi et al., 2018; Demirtas and Gunduz, 2021; Torrens et al., 2021). Thus, it is vital to identify new HCC-specific biomarkers, extensively study the pathogenesis of HCC and explore new precise therapeutic targets.

In recent years, with the in-depth understanding of cell death, a growing number of studies have confirmed that a serious of programmed cell death (PCD) programs, such as autophagy, ferroptosis and pyroptosis, play an indispensable role in tumorigenesis and progression (Mou et al., 2019; Li et al., 2020; Hou et al., 2020). Cuproptosis, as a new PCD program, has the characteristics of copper dependence and copper regulation. The mechanism is mainly through copper binding directly to lipoylated components in the tricarboxylic acid (TCA) cycle to mediate lipoylated protein aggregation and iron-sulfur cluster protein loss, which ultimately leads to proteotoxic stress and cell death (Tsvetkov et al., 2022). Copper has the functions as a metabolic cofactor at the active site as well as a dynamic signaling metal and metalloallosteric regulator, which is connected with various clinical diseases, especially cancer, because the growth and metastasis of tumors have higher requirements for this metal nutrient (Ge et al., 2022). As the Mortada study found, the copper levels in the plasma and bladder tissue were significantly higher in patients with bladder cancer than in those non-bladder cancer patients (Mortada et al., 2020). Interestingly, Atakul et al. (2020) found that the serum copper level of patients with endometrial cancer was significantly lower than that of normal people, and it was negatively correlated with the degree of tumor invasion. Relevant studies have shown that unbalanced copper homeostasis can induce many forms of cell death including apoptosis, autophagy and ferroptosis, through various mechanisms, such as reactive oxygen species accumulation, proteasome inhibition and mitochondrial dysfunction (Jiang et al., 2022). However, the specific mechanism of copper ion-induced PCD is still unclear. In addition, with the role of copper in tumor proliferation, invasion and metastasis, its antitumor potential has also been highlighted. Li et al. (2020) showed that the combination of disulfiram with copper can greatly improve its antitumor efficacy. Similarly, Mariani et al. (2021) also demonstrated that the combined use of copper complexes and cisplatin enhanced the antitumor effect against melanoma, lung cancer and breast cancer. Therefore, this could be a new strategy for cancer treatment by using copper ion metal carriers to eliminate cancer cells. However, the role of copper-induced cuoproptosis in HCC has not been reported. Therefore, this study aimed to explore the possible molecular markers and drug targets of copper death in HCC, comprehensively analyze the multiomics characteristics of cuproptosis related genes (CRGs), including genomics, transcriptomics and tumor microenvironment (TME), and extensively investigate the latent function of cuproptosis in the TME, clinical characteristics and prognosis of HCC to provide a new strategy and prediction model for clinical diagnosis, treatment and prognosis evaluation.

## 2 Methods and materials

### 2.1 Data sources

The workflow chart of this study is shown in Supplementary Figure S1. We downloaded 424 HCC patients with ribonucleotide (RNA) sequences, clinical information and GSE76427, GSE52018, GSE149614 datasets from the Cancer Genome Atlas (TCGA) (https://portal.gdc.cancer.gov/) databases and Gene Expression Omnibus (GEO) (https://www.ncbi.nlm.nih.gov/geo/). All raw files were normalized and annotated by using “limma” package in R, and the RNA sequences of fragments per kilobase million (FPKM) in TCGA were converted to transcripts per kilobase million (TPM) sequences. Then, batch effects of the three datasets were eliminated by the “Combat” algorithm. After integrating all datasets and excluding patients lacking overall survival (OS) data, the clinical data of 526 HCC patients were saved for succeeding analysis.

### 2.2 Difference analysis and cluster analysis of CRGs

We obtained 10 CRGs from previous publications [13]. According to the expression levels of CRGs in the HCC genomic dataset, the R language “reshape2” and “ggpubr” packages were used to analyze and plot the expression differences of CRGs in tumor and normal samples. Then, Kaplan‒Meier survival curves and interaction networks were analyzed and plotted using the HCC clinical dataset. Finally, according to the gene expression levels of CRGs, consensus unsupervised clustering (CUC) analysis was performed using the k-means method in the “ConensusClusterPlus” package to classify all HCC patients into different molecular subtypes. The principal component analysis (PCA) graph was drawn by analyzing the expression levels of CRGs and typing results by the “ggplot2” software package.

### 2.3 Difference analysis between CRG-related subtypes

We compared the prognosis and clinical characteristics of CRGS-related subtypes according to the clinical information files and classification results of HCC patients and drew Kaplan‒Meier survival curves and clinical characteristic heatmaps using R language for visualization. Then, we used the R language “GSVA” package to complete the gene set variation analysis (GSVA) enrichment analysis. At the same time, the ssGSEA algorithm in the “GSVA” package was used to quantitatively analyze the immune cells to compare the immune infiltration fraction between different subtypes.

### 2.4 Difference analysis between DEG-related subtypes and functional annotation

At first, we used R language “limma” package with parameters set to fold-change of 1.5 and adjusted *p*-value of <0.05 to extract CRG-related genes. Gene ontology (GO) and Kyoto encyclopedia of genes and genomes enrichment (KEGG) functional enrichment analyses were performed. Then, univariate cox regression analysis was performed on CRG-related genes to screen for prognosis-related differentially expressed genes (DEGs). The prognostic and clinical features between DEG-related subtypes were compared using the R “survival” package and the “PheATmap” package and visualized using Kaplan‒Meier survival curves and heatmaps. In addition, the differences in the expression of CRGs among the related types of CRG-related genes were compared again using the R packages “Reshape2” and “GGPubR.”

### 2.5 Prognosis model construction and validation

First, 526 HCC patients were randomly divided into training and test cohorts with 263 cases each according to the 1:1 ratio. The R package “Glmnet” was used to include prognosis-related DEGs in the Least Absolute shrinkage and Selection Operator (LASSO) and multivariate Cox regression analyses. Then, risk scores (RSs) were calculated for genes with nonzero regression coefficients. The RS was calculated as follows: RS = $\sum_{j=1}^{n}Xj*Coefj$ , (n represent number of prognosis-related DEGs, Xj and Coefj represent the DEGs expression level and risk coefficient). According to the median value of RS, two cohorts were divided into low-risk and high-risk groups, respectively. “ggalluvial” and “dplyr” are used to draw an alluvial diagram to visualize the model building process. Based on the typing results, RS differences were compared in CRG-related subtypes and gene-related subtypes using the “limma” and “ggpubr” packages, respectively. In addition, boxplots of the differential expression of CRGs and immune checkpoints were constructed by the “GGPLOT2” and “GGPUBR” packages. In addition, the survival differences and the receiver operating characteristic (ROC) curves were analyzed and plotted in the training and test cohorts.

### 2.6 Establishment of a nomogram scoring system

Based on the clinical data and grouping results, the “pheatmap” package was used to visualize the correlation of RS with survival status and the difference in expression of prognosis-related DEGs. Then, the “rms” package was used to construct a nomogram scoring system for predicting prognosis, and the “calibrate” function was used to draw a calibration curve for evaluating the accuracy of the prognostic prediction model.

### 2.7 Analysis of immune cell infiltration, TME and CSCs

The 22 immune cell infiltration degrees of each sample were evaluated by the “preprocessCore” and “e1071” packages, and then the “CiberSort” algorithm was used to calculate the correlation between immune cell infiltration degree and RS- and prognosis-related DEGs. Next, we used the “estimate” package to calculate the stromal score, immune score and estimation score and further evaluated tumor purity. In addition, we evaluated the relationship between CSCs and RSs using cancer stem cell (CSC) score files.

### 2.8 Somatic mutation and drug susceptibility analysis

Differences in somatic mutations of HCC patients were analyzed using the “maftools” package and displayed in a waterfall diagram. We continued to use the “pRophetic” software package and the genomics of drug sensitivity in cancer (GDSC) database (https://cancerrxgene.org) to calculate the semi-inhibitory concentration (IC50) value of commonly used chemotherapy drugs in tumors, and the Wilcoxon signed rank test was used to compare the difference in IC50 values between the risk group and the low-risk group. The filter condition was set to *p* < 0.001.

### 2.9 Western blotting

We randomly collected tumor tissues and paracancerous tissues from 3 HCC patients treated at the First Affiliated Hospital of Anhui Medical University from March to April 2022. Tissue and normal LO2 hepatocytes and Hep3b, Huh7 and LM3 HCC cells were extracted with RIPA (Beyotime, Shanghai, China) after grinding and filtration. Then, the tissue was lysed on ice and centrifuged. We collected the supernatant and then the protein concentration was determined by a BCA kit (Beyotime). After gel preparation, electrophoresis, and transfer, the membrane was incubated with primary (BEX1, 1:5000, 12390-1-AP, Proteintech), (G6PC, 1:5000, 66860-1-Ig, Proteintech), (NEIL3, 1:5000, 11621-1-AP, Proteintech), (GCLM, 1:5000, 66808-1-Ig, Proteintech), (NT5DC2, 1:5000, YLS-K0806, Yilisa) and secondary antibodies (Anti-rabbit IgG, HRP-linked Antibody, 7074S, CST). For Western blotting analysis, the proteins underwent separation by SDS‒PAGE, nitrocellulose membrane transfer, quick blocker kit (Beyotime) blocking. After that, the bands were detected by ECL Plus (EMD Millipore, Billerica, MA, United States).

### 2.10 Single-cell analysis

GSE149614 raw data were processed in R using the “Seurat” package, and cells within the tissue were filtered and visualized with parameters of 500–6,000 expressed genes and mitochondrial ratio >10%. After performing quality control (QC) and selecting cells using the “CreateSeuratObject” algorithm. Then, we used the Seurat function of FindIntegrateionAnchors to merge sample files with common anchors among variables (dims = 1:20). We performed PCA on the scaled data using the “JackStraw” algorithm and ranked the PCA using the “ElbowPlot” function. The FindClusters function was used to perform T-distributed stochastic neighbor embedding (tSNE) dimensionality reduction clustering (resolution = 0.2) on the first 20 PC data. The FindMarkers function of Seurat and the CIBERSORT algorithm were used to determine the expression of marker genes in each cluster.

### 2.11 Cell viability, migration and invasion assays

LM3 cells were purchased from. Then cells were cultured with RPMI-1640 supplemented with 10% FBS (Beyotime, Shanghai, China). Subsequently, two siRNAs were used to knock out the expression of BEX1. Specifically, the sequences of siRNAs for BEX1 were listed. Then, LM3 cells were cultured until the density reached about 60%–70%, and plasmids, shRNA and Lipofectamine™ 2000 diluents were prepared by serum-free Opti-MEM medium to prepare transfection complexes. The transfection complex was added to LM3 cells and cultured for 48 h. Then 5,000 cells with BEX1 siRNAs were cultured in 96-well plate and incubated for 48 h at 37°C. Then the cell viability was measured using CCK-8 kit. In addition, 24-well inserts were used to perform cell migration and invasion assays. Briefly, LM3 cells were infected with BEX1 siRNAs for 48 h. Then 5,000 cells were seeded in the upper chamber with (migration assay) or without Matrigel (invasion assay). Meanwhile, 500 μl fresh medium were transferred to the low chamber and incubated for 24 h. After that, cells were fixed by 3.7% paraformaldehyde and stained with crystal violet. Cell images were obtained by fluorescence microscope and counted using ImageJ.

### 2.12 Molecular docking

We used AutoDock Vina software for molecular docking (Trott and Olson, 2010). Sorafenib was used as a ligand, and the key genes BEX1, NEIL3, GCLM, G6PC and NT5DC2 were used as receptors. The PDB format files were downloaded from the RSCB PDB database (http://www.rcsb.org/). Convert the Suolafeini PDB format to MOL2 format using Chem3D. Then, AutoDockTools 1.5.6 (https://autodock.scripps.edu/) was used to process receptor proteins and small molecule ligands and saved as PDBQT format files. During the molecular docking process, the Lamarckian algorithm was used to identify the most binding mode. The search space volume was > 27,000 A^^3^, the exhaustiveness was set to 8 and the maximum number of conformations output was set to 15.

### 2.13 Statistical analyses

The study was carried out under the R version 4.1.3, Strawberry Perl version 5.32.1.1, GraphPad Prism 7 and ImageJ. *, **, *** indicated *p* < 0.05, *p* < 0.01, *p* < 0.001, respectively. A *p* < 0.05 was considered statistically significant.

## 3 Results

### 3.1 Differential expression of CRGs and identification of cuproptosis-related subtypes

**FIGURE 1:**
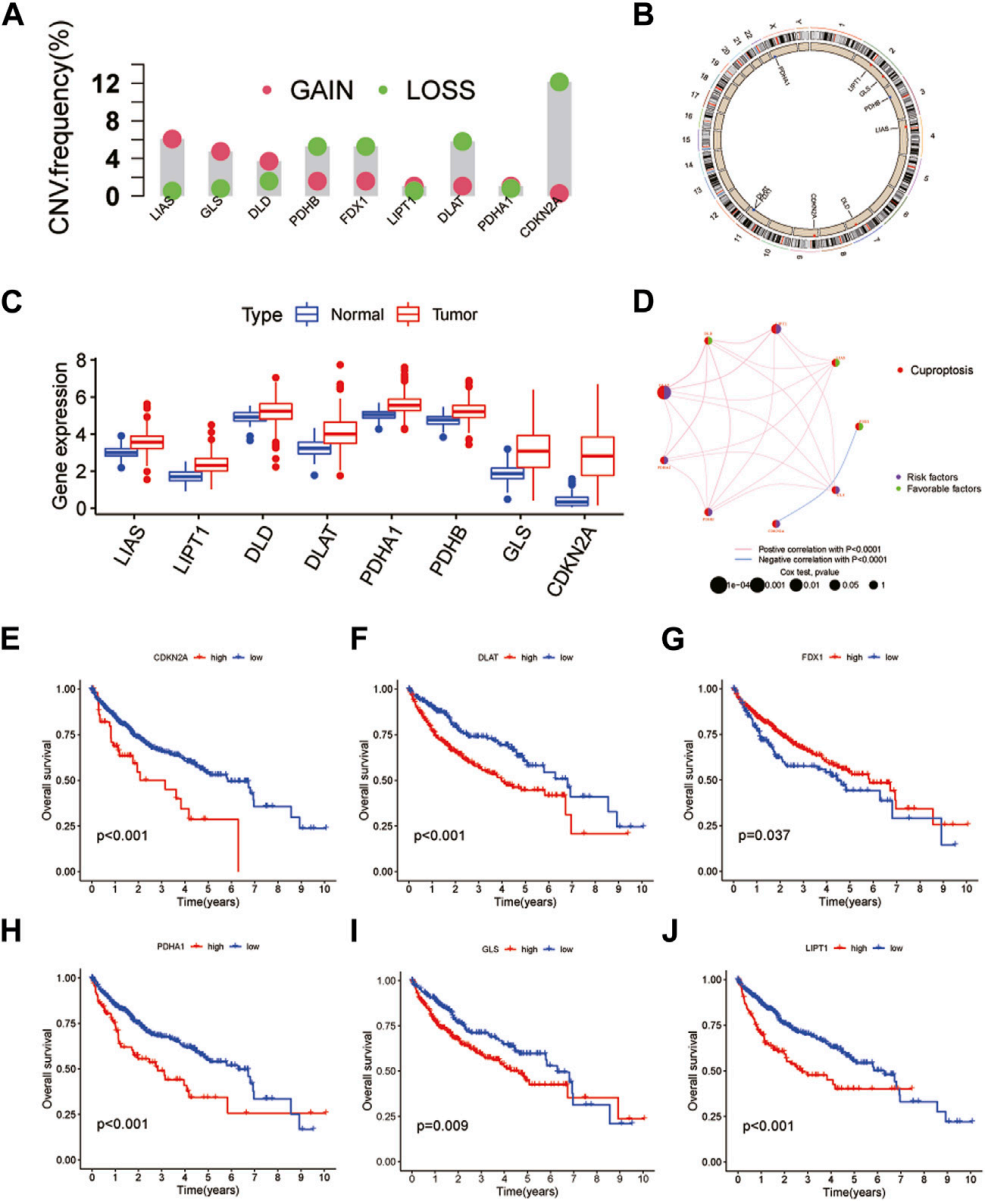
Genetic and transcriptional alterations of CRGs in HCC. **(A)** CNV of CRGs in 424 samples in TCGA. **(B)** Chromosomal localization of CRGs with CNV. **(C)** Expression of CRGs. **(D)** CRGs interaction network. **(E–J)** Kaplan-Meier survival analysis of 6 CRGs associated with HCC prognosis.

A total of 10 CRGs were obtained in this study for subsequent analysis. First, the results of copy number variation (CNV) analysis showed that there were clear CNVs in all genes except MIF1. CDKN2A had the highest gene mutation frequency and was mainly amplified on chromosome 9. The remaining CRGs also had different degrees of deletion and amplification variation, and their positions on the chromosome were also shown (Figures 1A, B). In addition, we obtained results showed that the remaining CRGs, except FDX1 and MIF1, were significantly highly expressed in tumor tissues. Interestingly, CDKN2A, which had the highest frequency of CNVs, also had the most significant differences in expression (Figure 1C). This suggests that the expression levels of genes in tumor tissues may be regulated by their CNVs. The results of the interaction network showed that CDKN2A and FDX1 were negatively correlated and were not found to be associated with other CRGs, while other genes interacted closely. Among them, FDX1, DLD and LIAS may be anticancer factors (Figure 1D). Further survival analysis found that CDKN2A, DLAT, FDX1, PDHA1, GLS and LIPT1 were associated with HCC prognosis (Figures 1E–J). Patients with highly-expressed FDX1 had a better prognosis, which further verified the anticancer effect of FDX1. The remaining five genes are the opposite. In addition, HCC patients were classified by CUC analysis of CRGs. The obtained results showed that k = 2 was the best choice, and the correlation between samples in subtypes A and B was the highest (Supplementary Figure S2). Besides, HCC samples can be separated into subtype A and subtype B, which was also verified by PCA (Supplementary Figure S3).

### 3.2 Differences in clinical features and functional annotations of CRG-related genes across subtypes

**FIGURE 2:**
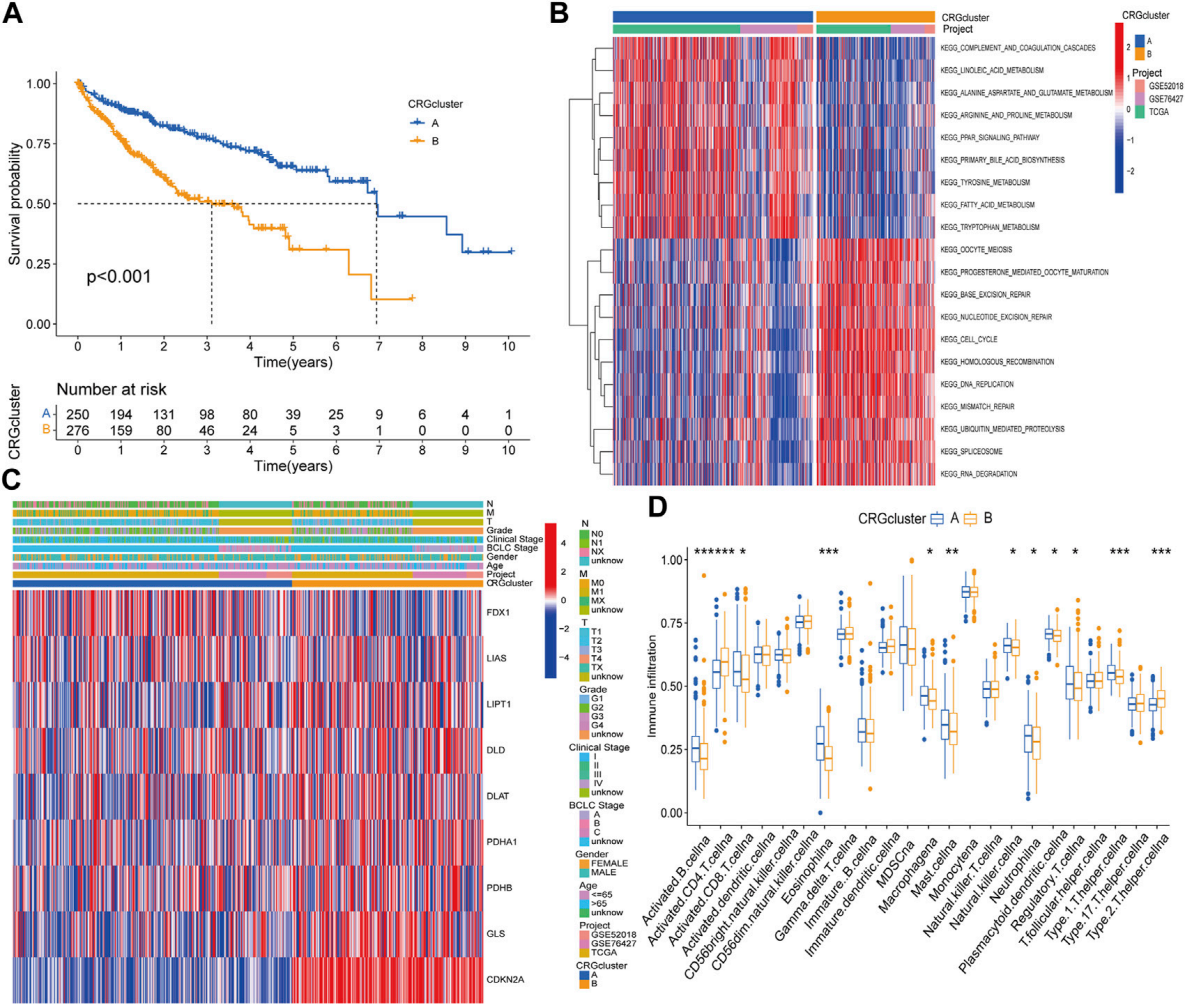
Difference analysis in distinct subtypes and functional annotations of CRGs related genes. **(A)** Survival curves between CRGs subtypes. **(B)** Clinical characteristics of CRGs subtypes. **(C)** GSVA enrichment analysis. **(D)** Differences in immune cell infiltration.

Kaplan‒Meier survival curves between different subtypes showed that patients with subtype A had a longer OS time than patients with subtype B (Figure 2A). Compared with subtype B, patients with subtype A had lower TNM stage and clinical stage than those with subtype B, the expression of FDX1 was relatively high, while the other genes were highly expressed in subtype B (Figure 2B). Moreover, GSVA suggested that subtype A was primarily enriched in fatty acid, bile acid and amino acid metabolism pathways, while cell cycle regulation, nucleic acid repair, nucleic acid synthesis and metabolism pathways were more likely to be found in subtype B (Figure 2C). The dysregulation of these pathways can cause gene mutation which can lead to abnormal cell metabolism or cell death. In the differences of immune cell infiltration. We found that activated B cell, activated CD8 T cell, eosinophilna, macrophagena, etc., degree was significantly higher in subtype A than in subtype B, whereas those of activated CD4 T cells and type 2 T helper cells were lower than those of subtype B (Figure 2D). To explore the underlying molecular behaviors of cuproptosis pattern, we identified 614 CRG-related DEGs and performed functional annotation analysis. These CRG-related DEGs were mainly enriched in biological processes such as cell cycle regulation and DNA replication (Supplementary Figure S4A). KEGG analysis showed that these genes were mainly enriched in cell cycling and DNA replication signaling pathways (Supplementary Figure S4B).

### 3.3 Identification of gene subtypes and construction of a prognostic model

**FIGURE 3:**
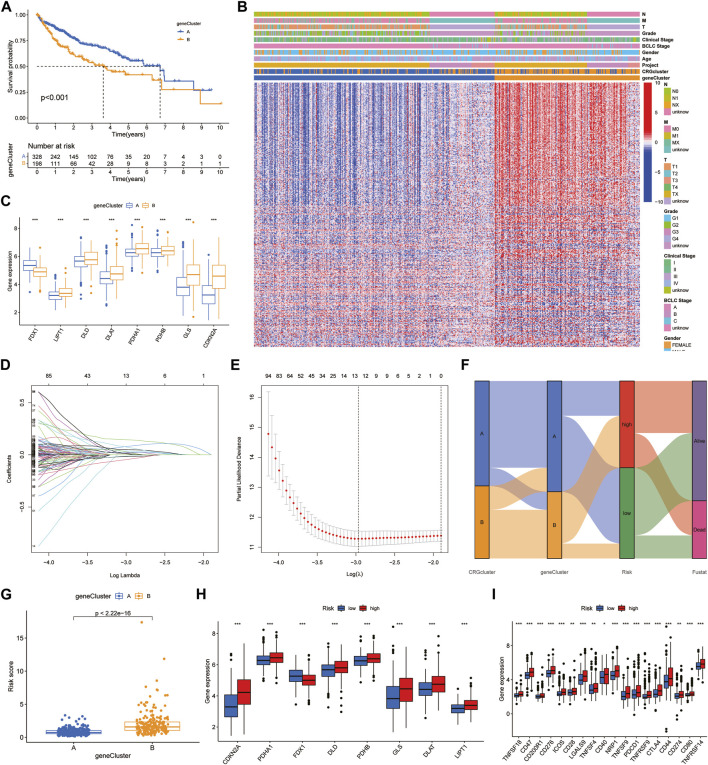
Identification of hub genes and construction of the prognostic model. **(A)** Survival curves between the hub genes subtypes. **(B)** Clinical characteristics of DEGs. **(C)** Expression of CRGs between DEGs subtypes. **(D,E)** LASSO regression analysis and partial likelihood deviance. **(F)** Alluvial diagram of subtype and RS distributions. **(G)** Differences of RS in DEGs clusters. **(H,I)** Expression of CRGs and immune checkpoints.

First, we obtained 382 DEGs that were associated with prognosis. We divided HCC patients into two subtypes, A and B, by CUC analysis (Supplementary Figure S5). Kaplan‒Meier survival curves showed that patients of subtype A survived longer than those of subtype B (Figure 3A). Combined with clinical characteristics, a positive correlation was found between B subtype pattern and advanced TNM staging, BCLC staging, and high expression of prognosis-related genes (Figure 3B). In addition, FDX1, DLD, CDKN2A, DLAT, PDHB, PDHA1, GLS and LIPT1 also showed significant differences in expression between the two subtypes. Among them, only FDX1 was highly expressed in subtype A, which further verified the role of FDX1 as an anticancer factor (Figure 3C). We then constructed a predictive model based on CRG-related genes. LASSO regression analysis was performed on CRG-related DEGs with prognostic value, and the risk coefficient of each CRG-related DEGs was evaluated. Thirteen prognostic CRG-related DEGs were retained according to the minimum partial likelihood deviance (Figures 3D, E). Then, we performed multivariate Cox regression analysis on 13 genes related to prognosis and finally found that BEX1, G6PC, GCLM, NEIL3 and NT5DC2 CRG-related genes are independent influencing factors of HCC. The Sankey diagram shows the distribution and correlation of patients with different prognoses in distinct subtypes and RS subgroups (Figure 3F). Among the different gene subtypes, the RS of subtype B was higher than that of subtype A (Figure 3G). Interestingly, the difference in CRG expression between the two groups was consistent with the difference between gene subtypes. Only FDX1 was highly expressed in the low-risk group, while DLD, CDKN2A, DLAT, PDHB, PDHA1, GLS and LIPT1 were all highly expressed in the high-risk group (Figure 3H). This further demonstrates the anticancer effect of FDX1. In addition, there was also a clear correlation between RS and immune checkpoints, including programmed cell death protein 1 (PD-1), programmed cell death 1 ligand 1 (PD-L1), cytotoxic T-lymphocyte-associated protein 4 (CTLA4) and cluster of differentiation 44 (CD44), which were highly expressed in the high-risk group (Figure 3I).

### 3.4 Validation of the prognostic model and development of a nomogram

**FIGURE 4:**
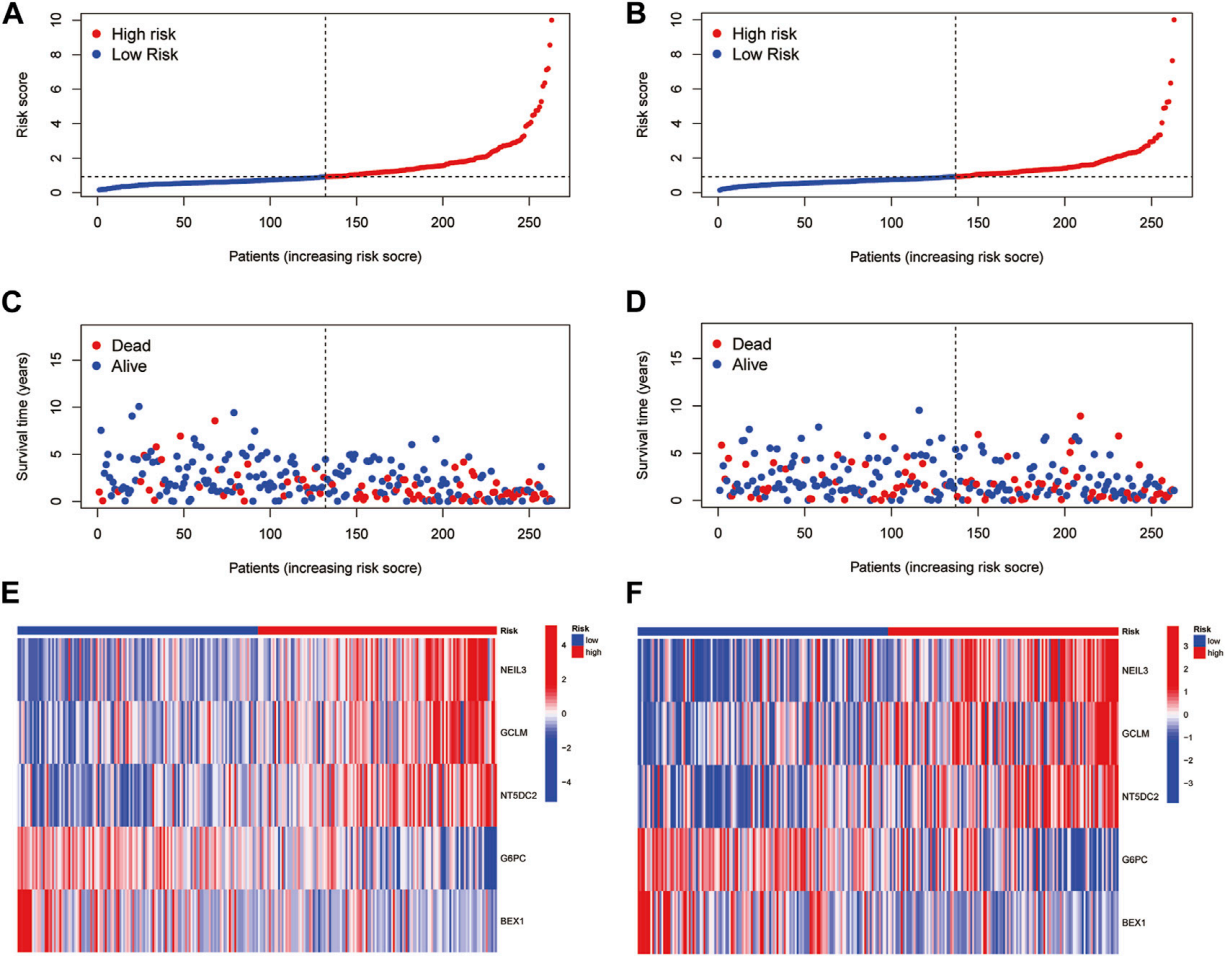
Prognostic value of the CRGs signature. **(A,B)** RS distribution. **(C,D)** Survival status. **(E,F)** Expression of the 5 hub genes.

**FIGURE 5:**
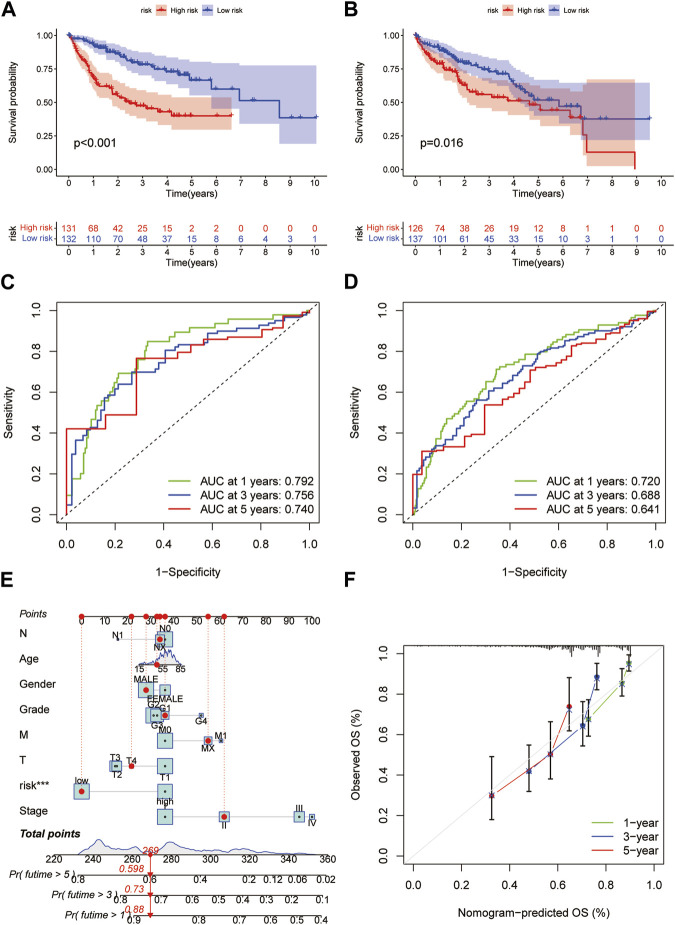
Prediction model and nomogram. **(A,B)** Kaplan-Meier curves in the training and test cohorts. **(C,D)** ROC curves estimate prognosis value. **(E)** Nomogram for predicting the OS of HCC patients. **(F)** Calibration curves of the nomogram.

Based on the median RS, the patients in the training and test groups were sorted according to the RS (Figures 4A, B). Survival status showed that patients in high-risk group of training group had a worse outcome than those in the low-risk group. We also found the similar results in the test group (Figures 4C, D). The heatmaps showed that NEIL3, GCLM and NT5DC2 had a higher expression level in the high-risk group, and G6PC and BEX1 were expressed at low levels (Figures 4E, F). Furthermore, in both the training and test groups, Kaplan‒Meier survival curves showed that the survival rate in the high-risk group was significantly lower than that in the low-risk group (Figures 5A, B). This may be related to the high expression of BEX1 and G6PC in the low-risk group, indicating that BEX1 and G6PC may have a synergistic effect with FDX1. In addition, to assess the efficacy of the prognostic model, the areas under the curve (AUCs) of the training group at 1, 3, and 5 years were 0.792, 0.756, and 0.740, respectively. Likewise, the test group also had the similar performance, with AUCs of 0.720, 0.688, and 0.641 at 1, 3, and 5 years, respectively (Figures 5C, D). Therefore, this study created a nomogram that integrated multiple molecular markers and clinicopathological parameters to predict the prognosis (Figure 5E). Meanwhile, the calibration diagram also demonstrated that the model had a perfect reliability (Figure 5F).

### 3.5 Correlation of RS with immune cells, TME score and somatic mutation and drug susceptibility analysis

**FIGURE 6:**
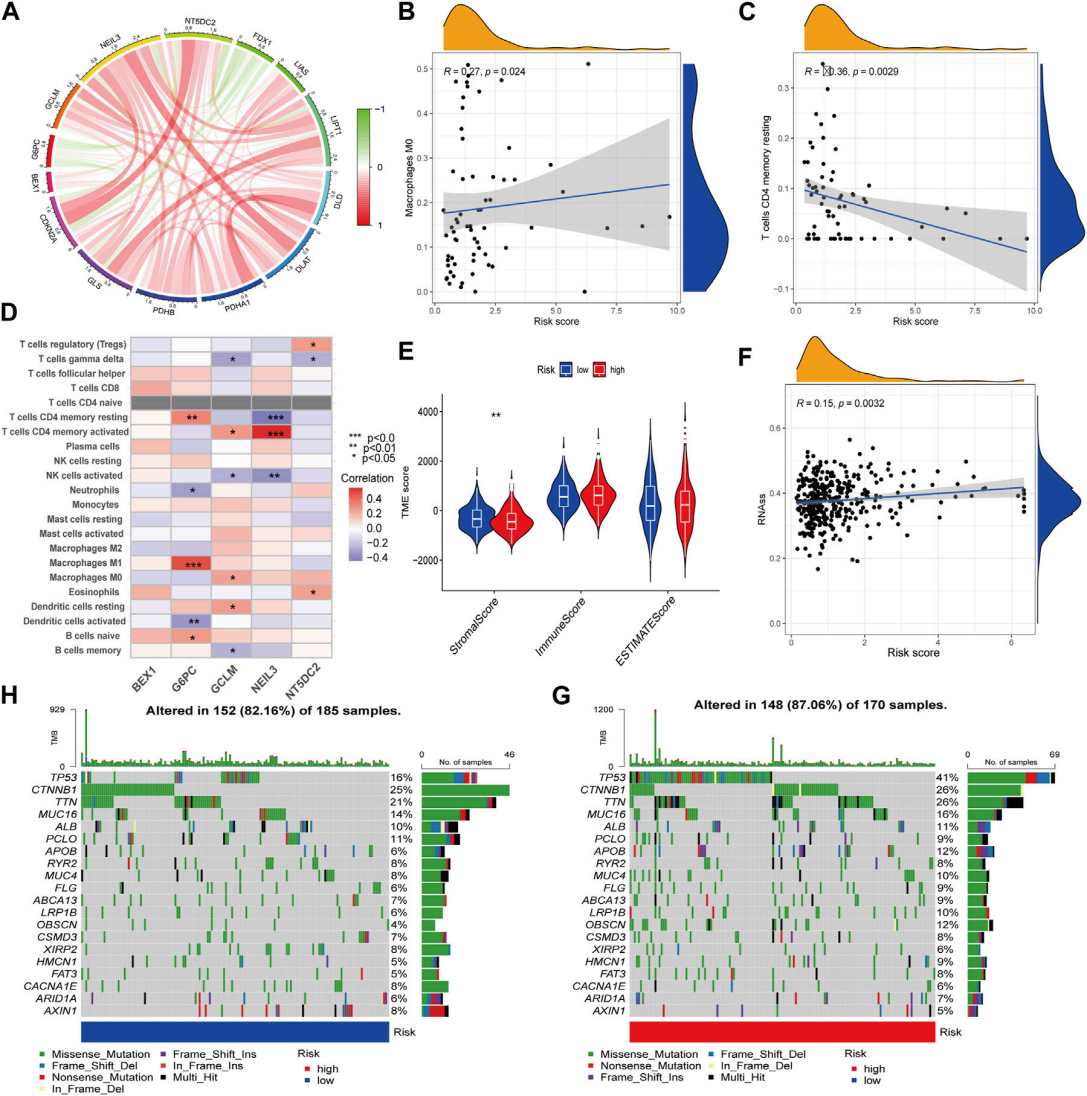
TME characteristics and drug susceptibility. **(A)**The interaction network of CRGs and hub genes. **(B,C)** Correlation between immune cells and RS. **(D)** Correlations between the immune cells and 5 hub genes. **(E)** Differences in the StromalScore and ImmuneScore. **(F)** Correlation of RS with CSCs. **(G,H)** Mutation of genes in distinct RS group.

The protein interaction network between CRG-related DEGs showed that five key prognosis-related genes in the prognostic model were negatively correlated with FDX1. Among them, NEIL3 and NT5DC2 were positively correlated with CDKN2A, and the correlation was the highest (Figure 6A). In addition, we found RS was positively correlated with M0 macrophages and negatively correlated with resting memory CD4^+^ T cells (Figures 6B, C). Among the five key prognosis-related genes, G6PC, GCLM, NEIL3 and NT5DC2 were significantly associated with a variety of immune cells, among which G6PC was positively associated with M1 macrophages, and NEIL3 was positively correlated with activated memory CD4^+^ T cells and negatively correlated with resting memory CD4^+^ T cells (Figure 6D). TME difference analysis found that the stromal score in the low-risk group was significantly higher than that in the high-risk group (Figure 6E). Our study also assessed the correlation between CSC and RS and concluded that RS was positively correlated with CSC (Figure 6F). Furthermore, we analyzed the somatic mutation profile. The obtained results showed that the top 10 mutated genes in the two groups were TP53, CTNNB1, TTN, MUC16, ALB, PCLO, APOB, RYR2, MUC4 and FLG. Among them, the PCLO mutation frequency was higher in the low-risk group, while other genes were lower than those in the high-risk group (Figures 6G, H). Finally, we evaluated the association between RS and drug sensitivity. We found that the IC50 values of various chemotherapeutics were significantly different between the high- and low-risk groups, including axitinib, gefitinib and erlotinib, sorafenib, vinorelbine, gemcitabine, nilotinib and tipifarnib (Supplementary Figures S6A–H).

### 3.6 BEX1 are differentially expressed in hepatocytes

**FIGURE 7:**
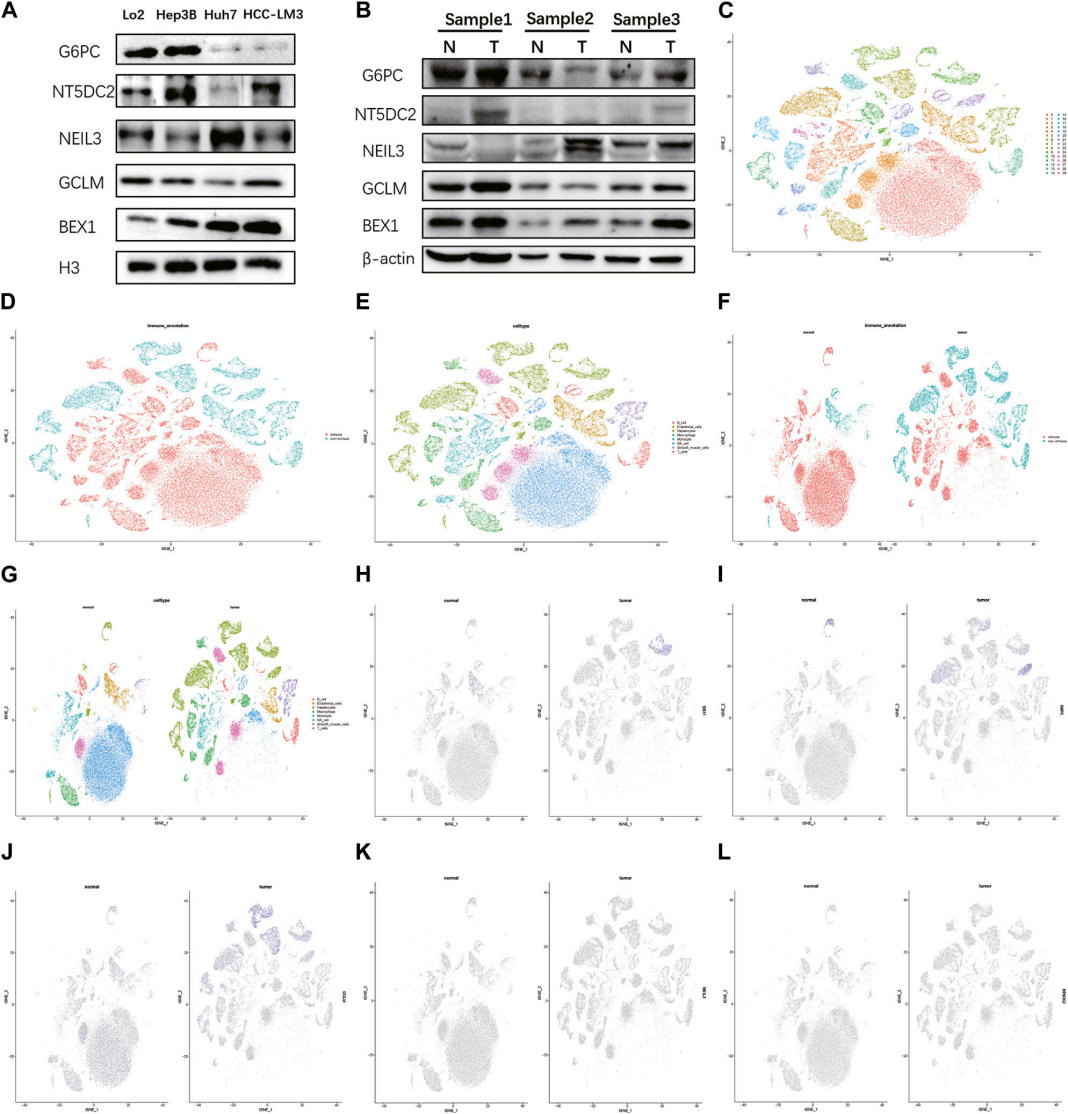
Western blotting and single cell analysis of the 5 hub genes. **(A,B)** Western blotting in HCC cell and tumor tissue. **(C–G)** Cell clusters and annotates for GSE149614 of HCC patients. **(H–L)** Expression pattern of 5 hub genes at the single-cell level in normal and tumor cell clusters through t-SNE analysis.

Detecting protein expression in HCC cells found that these five key genes were abnormally expressed to varying degrees in different HCC cells, especially BEX1 (Figure 7A). Compared with normal tissues, the expression levels of BEX1 in tumor tissues were significantly different, while the GCLM, G6PC, NEIL3 and NT5DC2 protein bands were unclear, which may be related to their low expression levels (Figure 7B). This was confirmed by single-cell analysis, which showed that there was no significant expression in hepatocytes (Figures 7K, L). To further explore the cellular expression of BEX1 in HCC tumors, 18 HCC samples in the GSE149614 dataset were analyzed. First, we controlled the effects of low-quality cells, mitochondrial genes, ribosomal genes, and hemoglobin. The correlations between the total number of unique molecular identifiers (UMIs) in each cell and the mitochondrial ratio, the total number of genes, and the hemoglobin ratio were 0.11, 0.91, and −0.01, respectively (Supplementary Figures S7A, B). No significant separation trend of HCC cells was observed when PCA was used to reduce the dimension, and we finally selected the top 20 PCs for further analysis based on the elbow plots (Supplementary Figures S5C, D). tSNE analysis classified HCC cells into 30 clusters (Figure 7C). The cells were annotated and divided into immune cell clusters and non-immune cell clusters (Figure 7D). We then proceeded to annotate eight subclusters using single-cell markers, including B cells, endothelial cells, hepatocytes, macrophages, monocytes, NK cells, smooth muscle cells and T cells (Figure 7E). In addition, normal and tumor tissues were similarly clustered and annotated by tSNE (Figures 7F, G). Finally, we tested the expression patterns of target genes in HCC cell clusters. The obtained results showed that the expression levels of BEX1 were the same as Western blotting, and they were only expressed in hepatocytes (Figures 7H–J). This finding indicates that BEX1 may be the key genes mediating cuproptosis in HCC cells.

### 3.7 Knockdown of BEX1 inhibited HCC cell proliferation, invasion and migration

**FIGURE 8:**
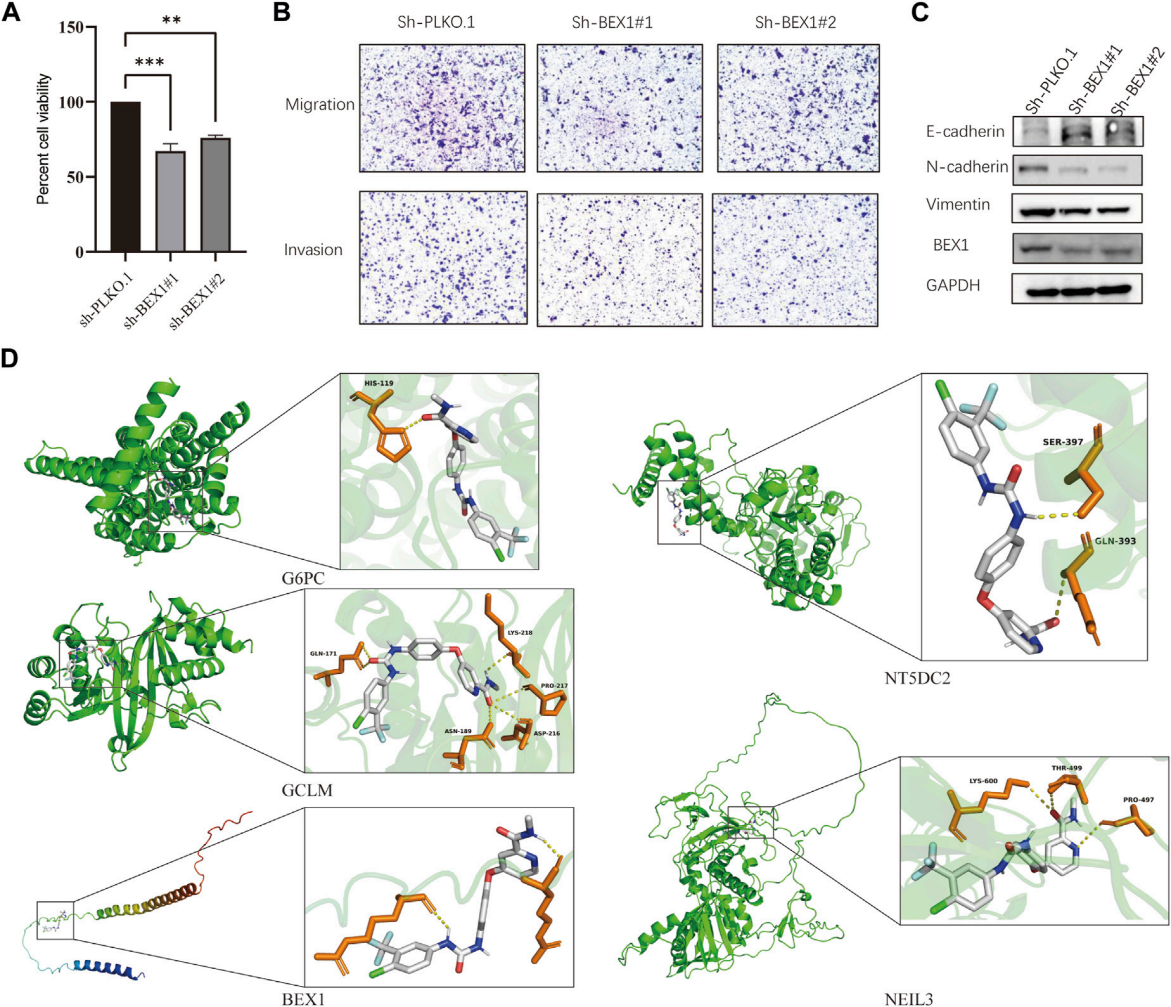
BEX1 biological function and molecular docking. **(A)** Cell viability experiment of BEX1 knockdown. **(B,C)** Transwell results and cadherin proteins expression in HCC cell with sh-BEX1. **(D)** Molecular docking of sorafenib and the 5 hub genes.

We found that BEX1 had the most significant differences in the five hub genes after detection of liver cancer cells, tissues and single-cell analysis. In order to further explore the biological function of BEX1, we used two methods to knockdown BEX1, both of which could effectively silence the expression of BEX1 (Figure 8C). The results of cell viability experiment showed that the viabilities of liver cancer cells decreased significantly after 48 h of BEX1 knockdown compared with the control group (Figure 8A). In addition, transwell results showed that the invasion and migration of LM3 cells with low BEX1 were significantly reduced (Figure 8B). Finally, we also detected the expression of epithelial mesenchymal transition (EMT) markers including E-cadherin, N-cadherin and Vimentin. The results indicated that BEX1 knockdown promoted E-cadherin expression and decreased the expression of N-cadherin and Vimentin. Which demonstrated that inhibition of BEX1 suppressed liver cancer cells EMT pathway (Figure 8C). Those results indicated that BEX1 deletion inhibited the proliferation, invasion and migration and EMT pathway of HCC cells.

### 3.8 Sorafenib specifically binds to key genes

To further explore whether the target gene may be a potential target of HCC immunotherapy, we used sorafenib as the ligand and the hub gene as the receptor for molecular docking analysis. The obtained results showed that the binding energies of sorafenib to G6PC, GCLM, BEX1, NT5DC2 and NEIL3 were −9.6, −8.5, −6.7, −8.3, and −8.0 kcal/mol, respectively. These hub genes can bind to sorafenib and form a stable conformation (Figure 8D). This finding suggests that the high expression of BEX1 in hepatocytes may be a potential therapeutic target to mediate cuproptosis in HCC.

## 4 Discussion

Numerous studies have indicated that PCD plays a key role in tumorigenesis and progression of HCC and antitumor response, with the powerful immunotherapeutic potential (Demirtas and Gunduz, 2021). However, the specific function in anti-tumor remains unknown, and research on cuproptosis is even scarcer. Therefore, this study extensively explored the copy number variation, clinical feature relationship, TME and immune infiltration of CRGs in HCC at the transcriptome level, single-cell level and network pharmacology level and revealed that GCLM and BEX1 may be potential therapeutic targets mediating cuproptosis in HCC.

CNV is a characteristic change in neoplastic diseases that has been gradually recognized in the postgenomic era. The human genome contains numerous repetitive sequences of varying frequencies and intensities, and CNV is defined as a type of alteration involving deletion, insertion, replication and multilocus variation of gene segments ranging from 1 Kb to 3 Mb (Kinross et al., 2012; Shi et al., 2021). Current studies suggest that CNV is not only the basis of individual genetic differences but also plays an essential role in tumorigenesis, invasion and metastasis. CNV-related indicators may become ideal tumor diagnostic markers (Behroozi et al., 2020; Pariyar et al., 2021). Huang et al. (2021) found that estrogen-related receptor alpha (ESRRA) CNV was significantly correlated with the histological grade of ovarian cancer (OC). The results indicated that CNV has the function in affecting biological phenotype and heterogeneity of tumors and promoting tumor progression. However, the above mentioned study evaluated only the CNV of genes alone and lacked a comprehensive study combining CNV and gene expression. In this study, we analyzed them together, and the characteristics of cuproptosis genes in HCC were more accurately identified. We found clear CNVs in all CRGs except MIF1. Among them, CDKN2A had the highest mutation frequency and expression difference, while FDX1 had no difference in expression between normal tissue and tumor tissue, although there was a higher amplification mutation. Similarly, previous studies by Ghaffari et al. (2016) showed that the CNV of the baculoviral inhibitor of apoptosis repeat-containing 5 gene is highly increased in tumor tissue and may become a marker for early cancer detection and prognosis. Therefore, this study suggests that CNV detection of these highly expressed CRGs may contribute to the diagnosis of HCC. Of note, CNVs can also activate proto-oncogenes and reduce the activity of tumor suppressor genes, thereby mediating the pathogenesis and prognostic mechanisms of various tumors, including liver cancer (Diskin et al., 2009; Nik-Zainal et al., 2016). In the analysis of prognosis and clinical features in this study, it was found that CNV genes were closely related to OS, disease progression and immune invasion in HCC patients. This finding indicates that CNV genes, such as CDKN2A, plays a key role in the escape from immune surveillance.

Furthermore, this study also screened CRG-related genes to construct an HCC risk model for predicting prognosis, searching for immunotherapy strategies and drug targets. The obtained results indicated that five CRG-related genes were independent prognostic factors of HCC. We further constructed a new prognostic model based on these genes. Compared with traditional TNM staging, this model integrates multiple molecular markers, clinicopathological parameters and other multilevel prognostic indicators, which can not only identify HCC patients with different risks but also more accurately and dynamically monitor tumor progression and prognosis. Similar studies have been conducted in previous studies, such as by Song et al. (2021), who screened pyroptosis-related genes and constructed a colorectal cancer (CRC) risk model. Based on these model results, Liao et al. (2022) found that FOXP2 promotes CRC pyroptosis by interacting with caspase-1. In addition, several studies have reported the role of cuproptosis in HCC. Zhao et al. (2022) explored cuproptosis related genes (CRGs) related to HCC survival and clinical features. And they found 20 CRGs were correlated to HCC outcomes and might be used as a prognostic biomarker for HCC. Besides, Zhang et al. (2022) investigated the association between FDX1 expression and cancer stages and outcomes in HCC. They constructed the model based on FDX1 and its related genes. And the AUC values of cuproptosis-related risk score (CRRS) in predicting the OS were 0.72 and 0.68 at 1 and 3 years respectively. Similarly, Yan et al. (2022) developed a predictive model (GCSH, LIPT1 and CDKN2A) based on CRGs in HCC. The AUC values of ROC analysis for 1 year OS were from 0.614 to 0.683. And they found LIPT1 might be a target in the treatment of HCC. In our study, a predictive model was constructed consists of five genes (BEX1, NEIL3, GCLM, G6PC and NT5DC2) through comprehensive bioinformatics analysis. The model was validated by an internal dataset with an AUC was 0.720–0.792 for 1 year, 0.688–0.756 for 3 years and 0.641–0.740 for 5 years, respectively, which showed a robust performance than previous models. Besides, molecular docking and experiments indicated that BEX1 may mediate the cuproptosis of hepatocytes as potential therapeutic targets for HCC. Therefore, the five key genes and risk models screened in this study play an extremely vital role in the diagnosis and treatment of HCC. On the other hand, the accuracy of quantitative analysis of these genes is not less than that of whole transcriptome sequencing, and it is more economical and clinically feasible.

With the great breakthrough in immunotherapy, an increasing number of researchers have realized that tumor cells do not exist in isolation, and the TME in which they are located plays an indispensable role in tumor progression. The TME is mainly composed of immune cells including lymphocytes, macrophages and granulocytes in the center and fibroblasts, inflammatory cells and various signaling molecules in the surroundings (Turley et al., 2015; Seager et al., 2017). Numerous evidence have shown that the cellular components in the TME are strongly linked to the progression, metastasis and efficacy of HCC (Chew et al., 2017; Satilmis et al., 2021). For example, Mano et al. (2019) found that bone morphogenetic protein 4 (BNP-4) could enhance the aggressiveness of HCC by activating fibroblasts (CAFs) to secrete cytokines in the TME. Besides, tumor-associated macrophages (TAMs) also exhibited different activation states in the TME depending on the different stimulus. Previous studies have shown that TAMs in HCC tumor stroma produce various proinflammatory cytokines, including TNF-α, IL-β, IL-6 and IL-23, which induce the expansion of CD4^+^ Th17 cells, according to the overexpression of PD-L1, CTLA4 to inhibit antitumor immunity (Kuang et al., 2010). Previous studies have mainly concentrated on the innate T cell immune response. However, in the ongoing exploration of advanced HCC and other cancers, immunotherapy methods increasingly focus on immune checkpoint inhibitors (ICIs), such as CTLA-4, PD-1 and PD-L1, which lock the immune checkpoint inhibition pathways (Kudo, 2019a). These results indicate that ICIs based on TME changes will be a new therapeutic strategy for HCC.

However, in this research, we found that multiple immune markers, such as PD-L1, CD276, CD80 and CTLA4, were significantly higher in the high-risk group with worse prognosis than in the low-risk group, and RS was significantly correlated with M0 macrophages and memory CD4^+^ T cells. This suggests that HCC hepatocytes may achieve immune resistance or immune escape by overexpressing PD-L1 and binding to PD-1 on the surface of specific cytotoxic T cells. Of course, this mechanism is more complicated in practice. For example, Kudo (2019b) found that activated CD8^+^ T cells released interferon gamma (IFN-γ) during the process of initial cellular immunity, which not only attacked tumor cells but also engaged with receptors on the surface of cancer cells to upregulate PD-L1 and inhibit antitumor effects. Currently, research on PD-1 and PD-L1 inhibitors is in full swing and has become an important part of the systemic treatment of HCC in clinical practice. Numerous studies demonstrated that nivolumab and pembrolizumab have shown efficacy in the treatment of HCC especially as an alternative strategy after sorafenib failure or unacceptable toxicity (El-Khoueiry et al., 2017; Zhu et al., 2018). In addition, as a transmembrane receptor on the surface of activated T cells, the expression of CTLA4 is strictly regulated. In resting or naive T cells, CTLA4 is mainly located in intracellular vesicles (Valk et al., 2008). However, on activated T cells, CTLA4 directly competes with CD28 for B7 ligands to mediate tumor immune responses (Hathcock et al., 1993). In addition, CTLA4 activation also supported the transformation of CD4^+^ T cells into regulatory T cells by increasing transforming growth factor-β (TGFβ) secretion and forkhead box protein 3 (FOXP3) expression (Zheng et al., 2006). In fact, many mechanisms are involved in the tumor immune response in the TME, but the specific effects are still controversial.

In addition, we detected the protein level of five important genes and found that the expression of BEX1 in tumor cells and tissues was higher than that in normal liver cells and tissues. To further explore the biological function of BEX1, cell viability experiment and transwell results showed that BEX1 deletion inhibited the proliferation, invasion and migration of HCC cells. These results suggest that BEX1 may play a key role in HCC tumorigenesis and development. However, only few studies described the role of BEX1 in HCC. BEX1 (brain-expressed X-linked protein 1), attached to the BEX family and consists of five proteins with unclear functions (Kazi et al., 2015). BEX1 was initially thought to be associated with retinoic acid differentiation in teratomas (Faria et al., 1998). Later, multiple research teams successively reported its expression changes in many cancers (Quentmeier et al., 2005; Foltz et al., 2006; Kazi et al., 2015). Current research suggests that in addition to being involved in the regeneration of neuronal axons and regulating the cell cycle, BEX1 is also involved in the proliferation and invasion (Vilar et al., 2006; Khazaei et al., 2010; Doi et al., 2020; Lee et al., 2021) showed that BEX1 and BEX4 can improve the tumor formation and radio resistance of glioblastoma multiforme cells. In breast cancer, overexpression of BEX1 and BEX2 can inhibit the apoptosis of tumor cells (Naderi et al., 2007). Deficiency of Bex1 expression led to the decrease of cell proliferation, colony and tumor formation, and the increase of cell apoptosis in acute myeloid leukemia (Lindblad et al., 2015). These studies all suggest that BEX1 may be an oncogene, but there is still a lack of studies on the gene, especially in HCC. Sagawa et al. (2015) showed that BEX1 was upregulated in Cx32ΔTg rat liver, and knockdown of BEX1 could significantly inhibit the growth of rat hepatoma cell lines. In human HCC studies, Wang et al. (2021) discovered that BEX1 is an oncofetal protein that interacts with RUNX family transcription factor 3 (RUNX3) in hepatoblastoma (HB) and CSC-HCC to block β-catenin transcription and activate the Wnt/β-catenin signaling pathway, thereby regulating the self-renewal of hepatic CSCs. These results indicate that BEX1 is a promising therapeutic target forHB and CSC-HCC, and targeting the BeX1-mediated Wnt/β-catenin signaling pathway may help to resolve the heterogeneity and high recurrence rate of HCC.

Previous studies also explored the potential targets of cuproptosis in HCC. For example, FDX1, LIPT1, DLAT, CDKN2A and GLS were reported to have potential value as cuproptosis targets in HCC. Zhang et al. (2022) found FDX1 was downregulated in HCC, and higher expression of FDX1 was related to a better outcome. Beside, FDX1 inhibited the proliferation and colony formation of tumor cells in the presence of copper ions and this inhibitory effect was diminished by using tetrathiomolybdate (TTM) to chelate copper ions, which indicated that FDX1 exerted anti-tumor effects through cuproptosis (Li et al., 2022). And Yan et al. (2022) revealed that LIPT1 was higher expressed in HCC and patients with low LIPT1 expression had longer OS than those high LIPT1 expression. Cell proliferation assay showed that LIPT1 depletion inhibited cancer viability in HCC cells. Besides, LIPT1 knock down significantly suppressed cell migration and invasion capacity in HCC cells. Similar results were found in DLAT (Yang et al., 2023), CDKN2A and GLS (Ma et al., 2023). In our study, we found BEX1 deletion inhibited the proliferation, invasion and migration and EMT pathway of HCC cells. Besides, BEX1 can bind to sorafenib and form a stable conformation which further suggests that the BEX1 in hepatocytes may be a potential therapeutic target to mediate cuproptosis in HCC.

Furthermore, we detected the protein level of five important genes and found that the expression of the other genes except BEX1 in tumor cells and tissues was higher than that in normal liver cells and tissues. To further explore the specific cellular expression patterns of these genes in HCC, we conducted scRNA-seq data to identify the distribution of G6PC, GCLM NEIL3 and NT5DC2 in different cell subclusters in HCC tissues including HCC tumor cells and non-tumor cells. And the results clearly revealed that GCLM and G6PC were only highly expressed in HCC cells, while lowly expressed in non-tumor cells. The expression of NEIL3 and NT5DC2 was not obvious in hepatocellular carcinoma cells. These results suggest that GCLM and G6PC regulate HCC development directly on the target of hepatocytes rather than other cells, which provides potential therapeutic targets.

Glucose-6-phosphatase (G6PC) is a component critical for catalyzing glycogenolysis, and the downregulated of G6PC enhances glucose storage in premalignant cells (Franco et al., 2005; Resaz et al., 2014), and glycogen accumulation is a key carcinogenic event in the malignant transformation of the liver (Liu et al., 2021). Bioinformatics analysis demonstrated that low expression of G6PC was related to poor outcomes in HCC (Tian and Liao, 2022). However, there still is an empty in studies related to the regulation of copper by BEX1 and GP6C.

GCLM is a glutamate cysteine ligase modifier subunit that is the main component of glutathione (GSH) synthetase and participates in the synthesis and metabolism of GSH on multiple levels, and increasing evidence has shown that GSH metabolic dysregulation is involved in the pathophysiological mechanisms of various diseases, including diabetes, liver fibrosis, alcoholic liver disease, and malignant tumors (Liu and Gaston, 2010; Lv et al., 2019; Shen and Wang, 2021). Previous studies have shown that HCC patients showed higher levels of oxidative stress markers and low levels of GSH and GSH-related antioxidant enzymes in plasma compared with nonalcoholic steatohepatitis patients (Shimomura et al., 2017). The imbalance between high oxidative stress and low antioxidant capacity may be an important reason for the occurrence and development of HCC. Therefore, regulating the expression of GCLM may indirectly play an essential function in the development of HCC. In addition, studies have shown that GCLM expression is mainly regulated by transcription factors, including activator protein-1 (AP-1), nuclear factor kappa B (NFκB) and nuclear factor erythroid 2 related factor 2 (Nrf2) (Jaiswal, 2004; Yang et al., 2005; Roos et al., 2020) confirmed that treatment of human hepatocellular carcinoma (HepG2) cells with the receptor tyrosine kinase inhibitor lapatinib activates the Kelch-like ECH-associated protein 1 (Keap1)-Nrf2 signaling pathway, thereby upregulating GCLM levels and inducing GSH synthesis. Most interestingly, Thai et al. (2021) found that copper nanoparticles (Cu NPs) have the most significant effect on NrF2-mediated cytotoxicity, and upregulated GCLM can be used as a biomarker for Cu NP exposure in HCC cells. This is similar to our study, indicating that GCLM is more sensitive to copper damage and may be a key target in mediating cuproptosis in hepatocytes.

Finally, we verified the potential role of these hub genes as therapeutic targets in HCC. We performed drug-target network and molecular docking analyses with sorafenib as the ligand and the hub gene as the receptor, and the obtained results showed that BEX1, G6PC and GCLM could bind to sorafenib and form stable conformations. Sorafenib, as a multiple-target tyrosine kinase inhibitor (TKI), has the functions of anti-angiogenesis and anti-proliferation, besides it can also prolong the overall median survival time of patients with advanced HCC (Llovet et al., 2008). Two important clinical trials, the Asia-Pacific and Sorafenib HCC Assessment Randomized Protocol (SHARP), also showed that sorafenib has a powerful function in improving the prognosis of patients with HCC (Cheng et al., 2009; Vogel and Saborowski, 2020). In addition, Sorafenib is an effective first-line treatment in patients with advanced HCC (Xing et al., 2021). *In vitro* experiments revealed that sorafenib inhibits tumor cell viability, promotes cell apoptosis in HCC cells (Xie et al., 2018). Therefore, we used sorafenib as the ligand and the hub gene as the receptor for molecular docking analysis.

Although current ICIs have opened up a new strategy in treating malignant tumors, sorafenib is still the first-line drug for HCC chemotherapy (Benson et al., 2021). In recent years, studies on the discovery of new efficacy and potential therapeutic targets for known drugs are not uncommon; Pushpakom et al. (2019) found that Raloxifene was initially used to treat osteoporosis and was later approved for the treatment of breast cancer. Additionally, a study by Pang et al. (2019) is similar to ours; they confirmed that C3 and ANXN1 can stably bind to Vorinostat, which can be used as potential therapeutic targets for papillary renal cell carcinoma through genetic screening and molecular docking. Therefore, it is reasonable to speculate that BEX1, G6PC and GCLM can be used as potential therapeutic targets of HCC to mediate the mechanism of cuproptosis in hepatocytes.

Nevertheless, there are still several limitations in this study. First, this is a prospective study, outcomes can be affected by the introduction of unknown variables during follow-up, or changes in known variables including treatment modality disease and surgery-related complications. Second, our prognostic model was obtained based on TCGA and GEO data but lacked validation with external datasets, limited the breadth of its use. Third, we investigated the cellular molecular function of BEX1 in HCC but did not explore the interaction between BEX1 and copper ions in hepatic tumor cells, therefore, we could not clarify whether the BEX1-regulated cellular activity was related to copper ions. Finally, the specific mechanism by which BEX1 regulates HCC progression remains unclear. In the future studies, external datasets or one’s own data to assess the stability of the model is needed. Furthermore, *in vivo* and *in vitro* experiments are needed to investigate the exact mechanism by which BEX1 regulates cuproptosis especially in the presence of copper ions or when activity is inhibited.

## 5 Conclusion

This study extensively explored the multi-omics features of CRGs in HCC, including CNV, clinicopathological indicators, prognosis, immune infiltration, and TME, and constructed a prognostic prediction model integrating multiple molecular markers and clinicopathological parameters, which offers a new method for clinical diagnosis and prognosis evaluation. Meanwhile, GCLM and BEX1 were identified as hub genes, which are potential therapeutic targets to mediate the cuproptosis program in HCC cells.

## Data Availability

The raw data supporting the conclusion of this article will be made available by the authors, without undue reservation.
