# Supplementary figures and images for: Single-cell and genetic multi-omics analysis combined with experiments confirmed the signature and potential targets of cuproptosis in hepatocellular carcinoma
Source: Front Cell Dev Biol. 2023 Sep 8;11:1240390. doi: 10.3389/fcell.2023.1240390 (PMC10516581; doi:10.3389/fcell.2023.1240390)

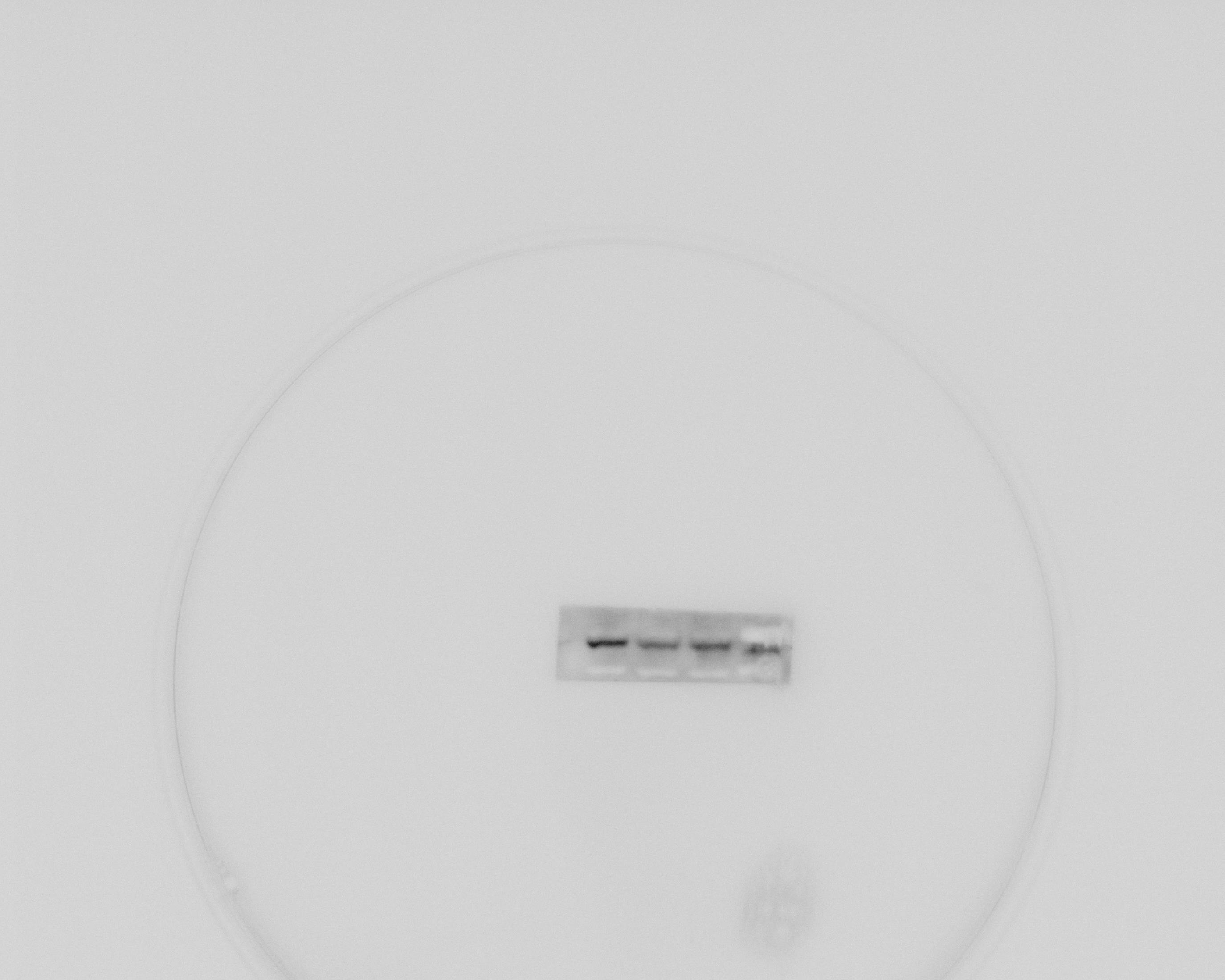

Supplement: Supplementary file 3 [file DataSheet2.zip › Raw data/CF-23.5.31/BEX1/BEX1.jpg]

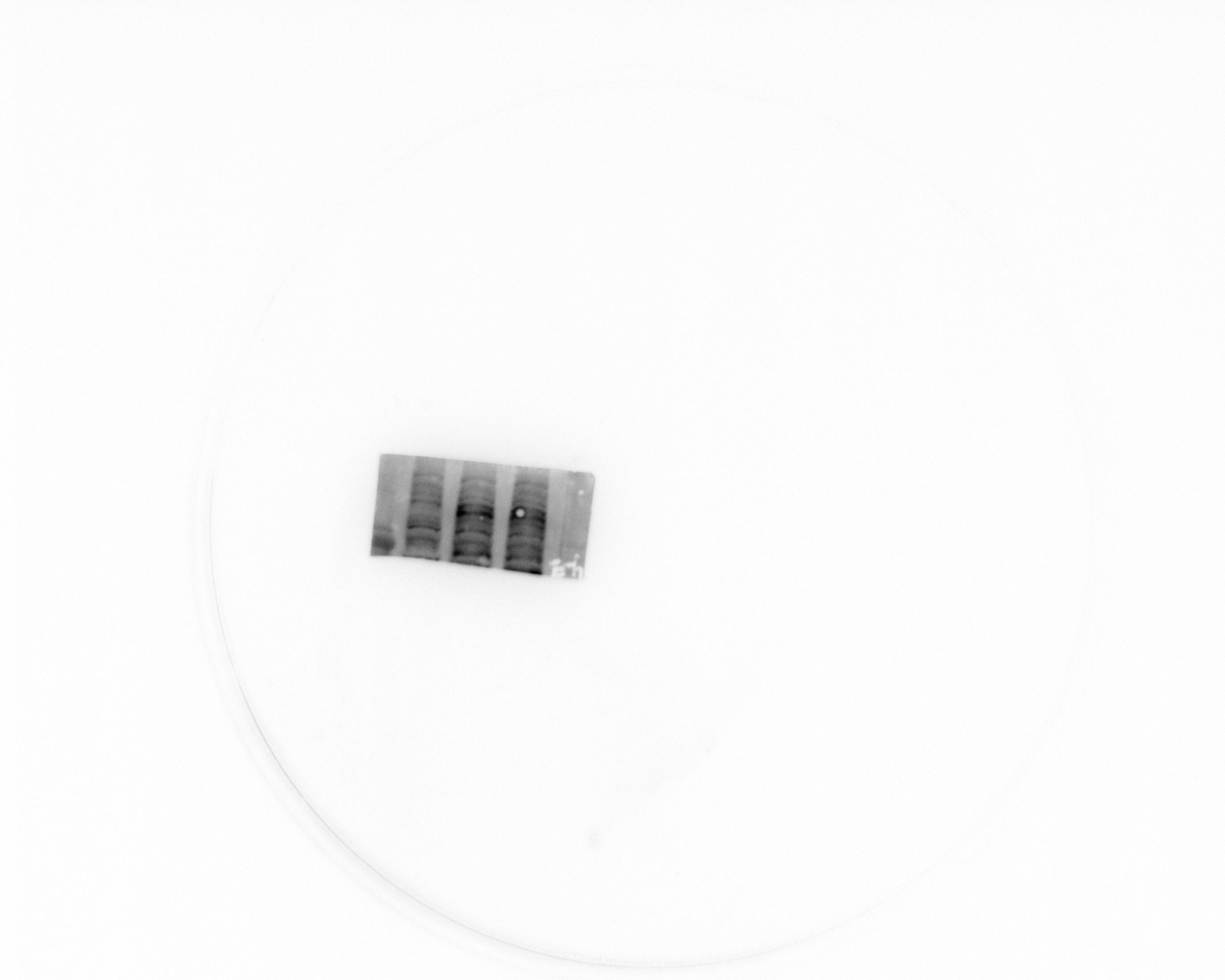

Supplement: Supplementary file 3 [file DataSheet2.zip › Raw data/CF-23.5.31/BEX1/E-CADHREIN-BEX1.jpg]

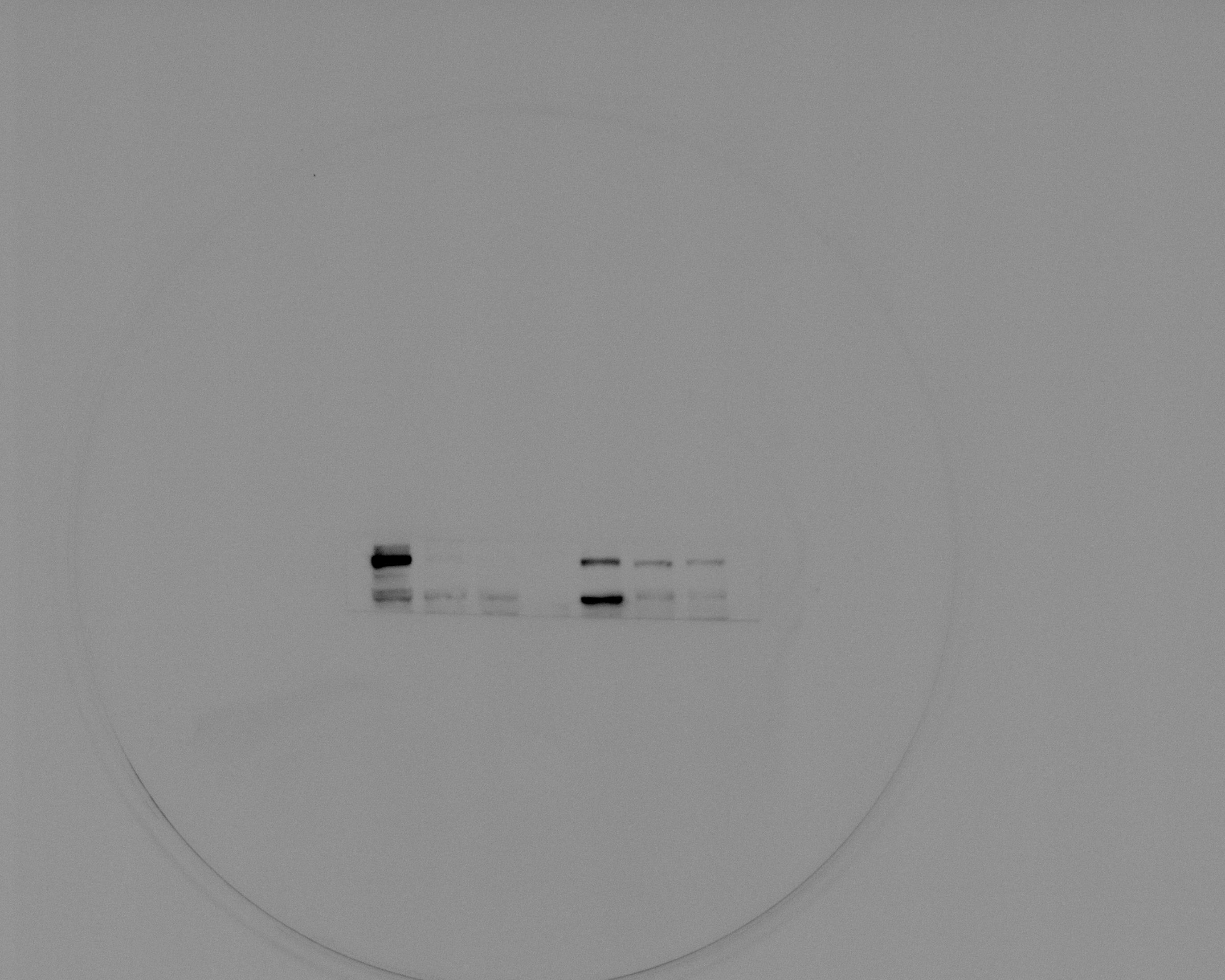

Supplement: Supplementary file 3 [file DataSheet2.zip › Raw data/CF-23.5.31/BEX1/N-CADHERIN-BEX1.jpg]

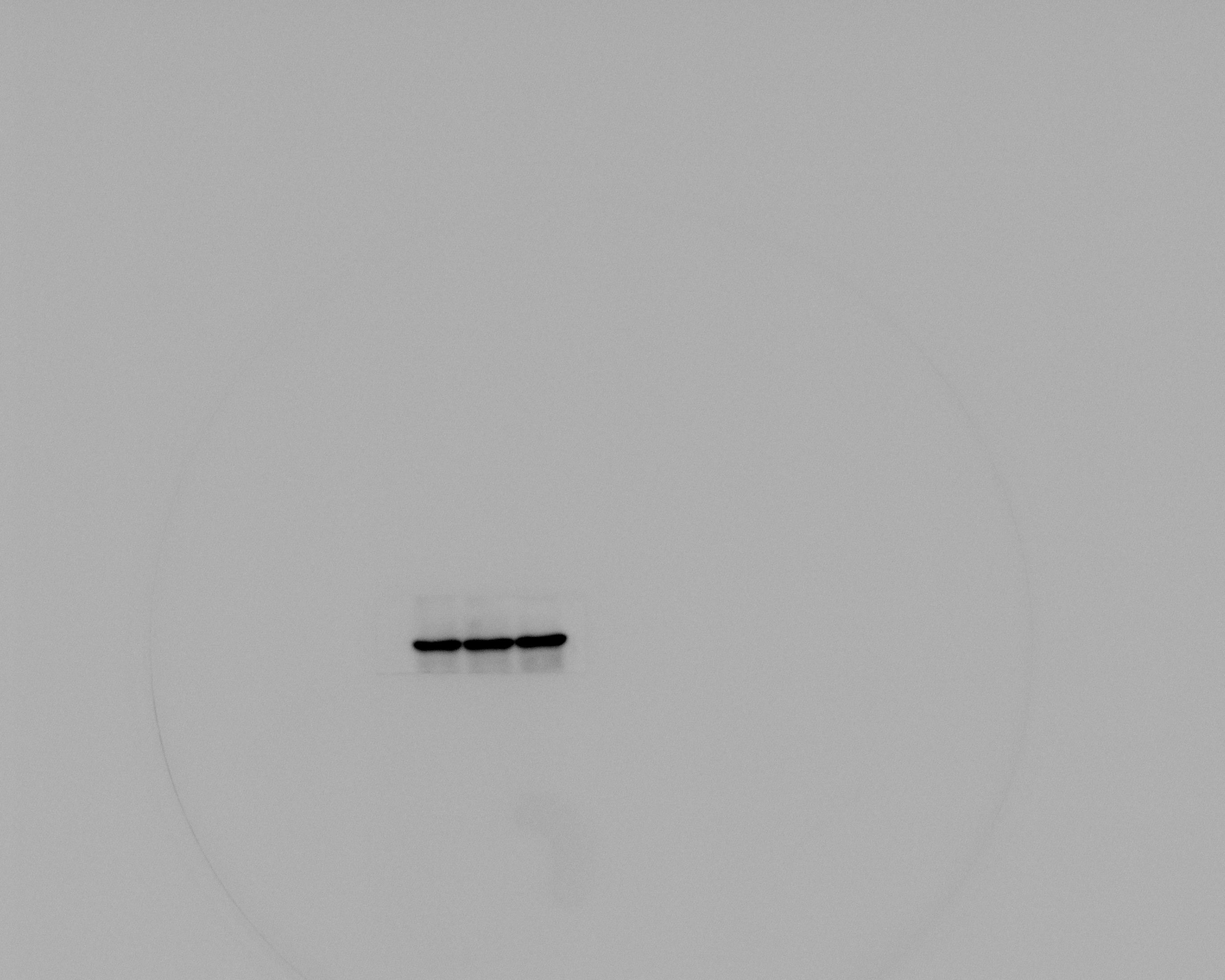

Supplement: Supplementary file 3 [file DataSheet2.zip › Raw data/CF-23.5.31/BEX1/gapdh-lm3.jpg]

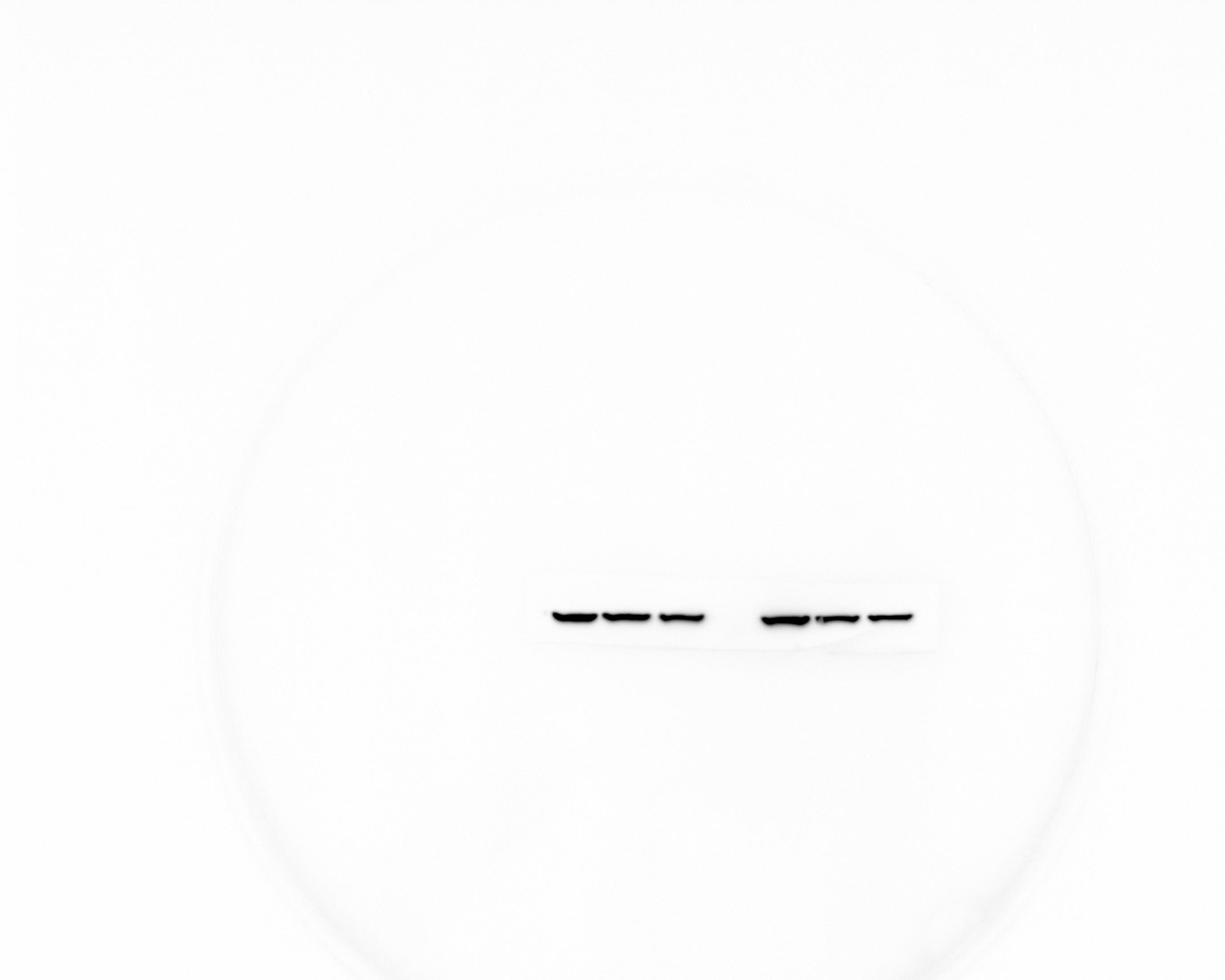

Supplement: Supplementary file 3 [file DataSheet2.zip › Raw data/CF-23.5.31/BEX1/vimentin-BEX1.jpg]

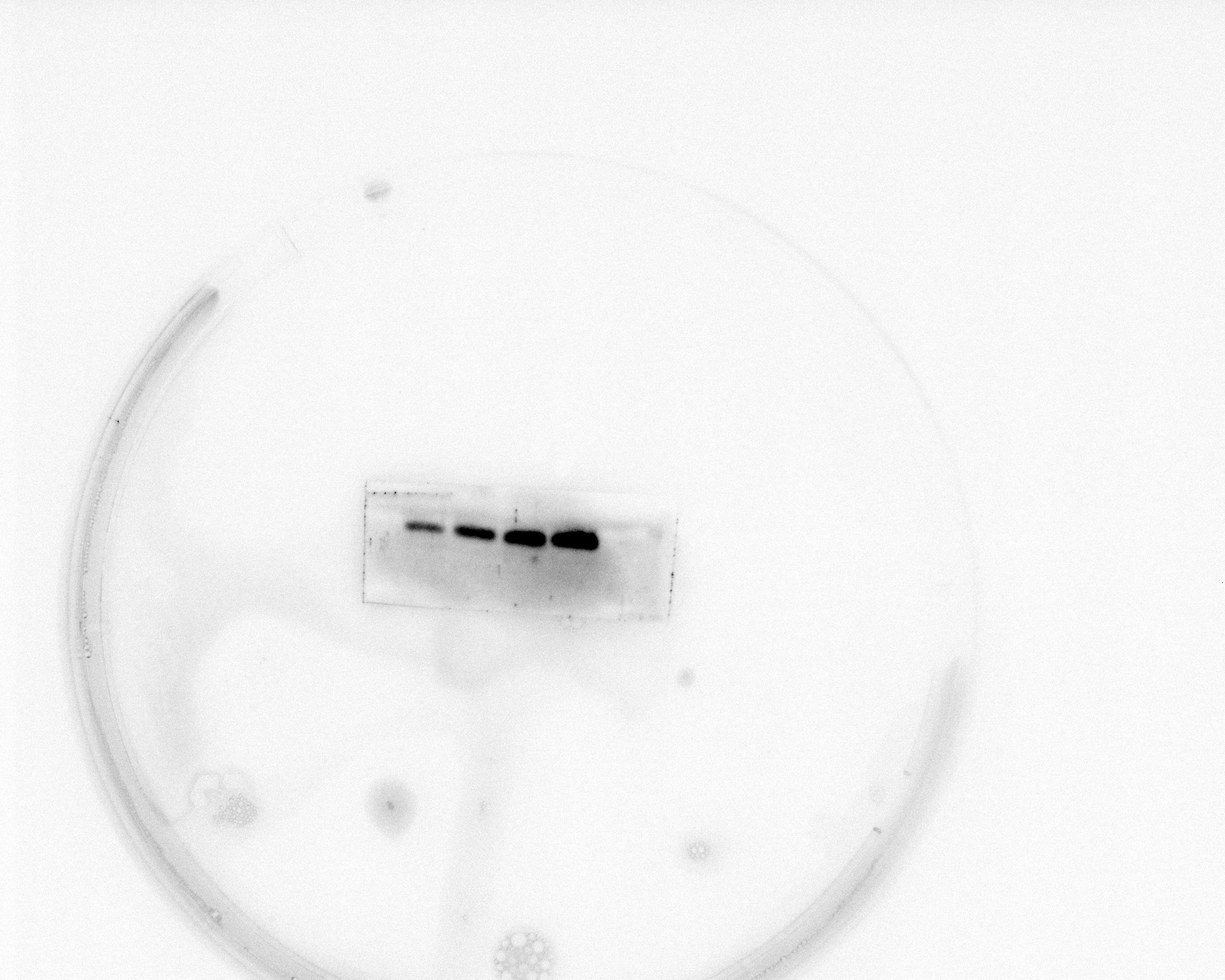

Supplement: Supplementary file 3 [file DataSheet2.zip › Raw data/CF-23.5.31/cell/BEX1-HCC.jpg]

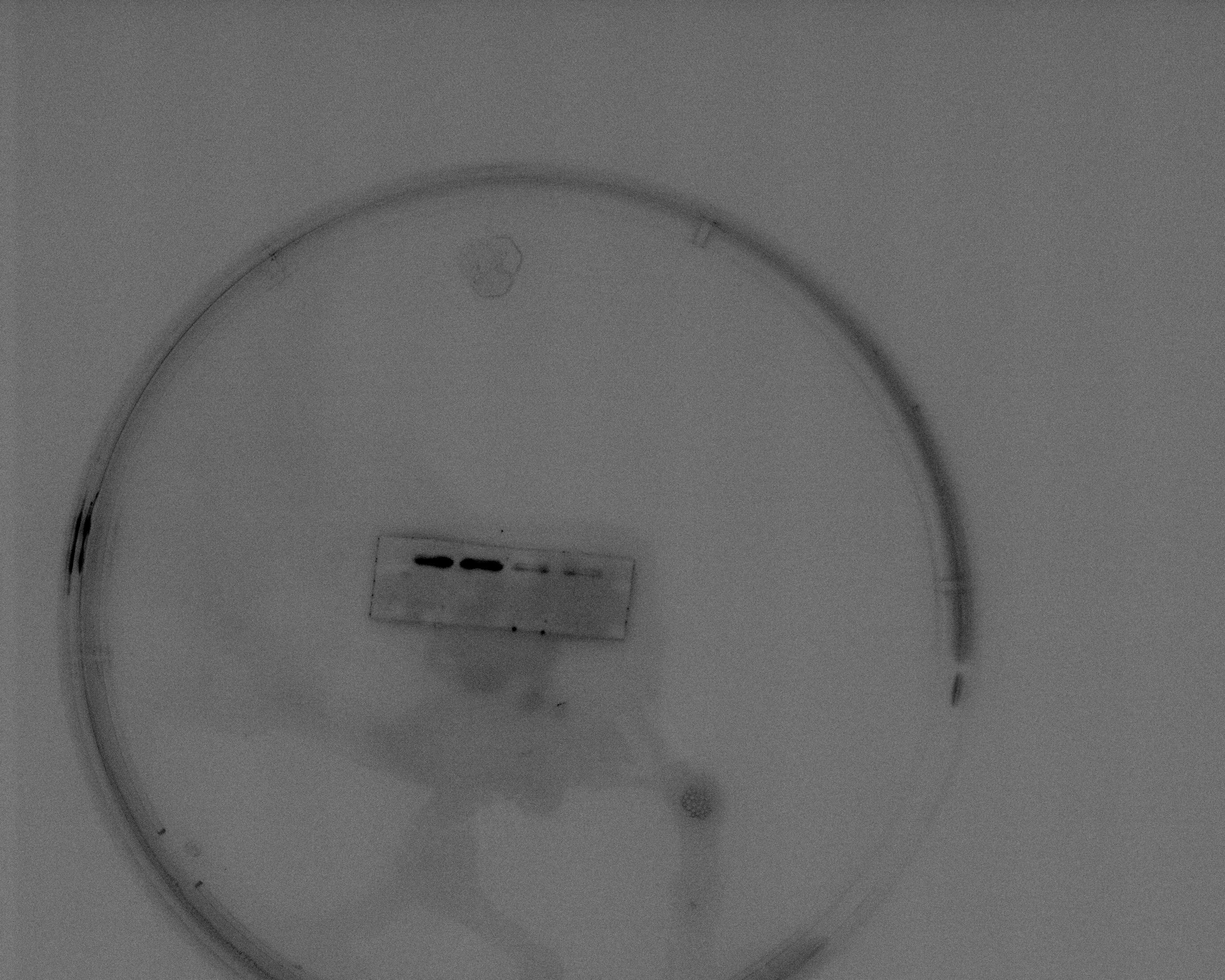

Supplement: Supplementary file 3 [file DataSheet2.zip › Raw data/CF-23.5.31/cell/G6PC-HCC.jpg]

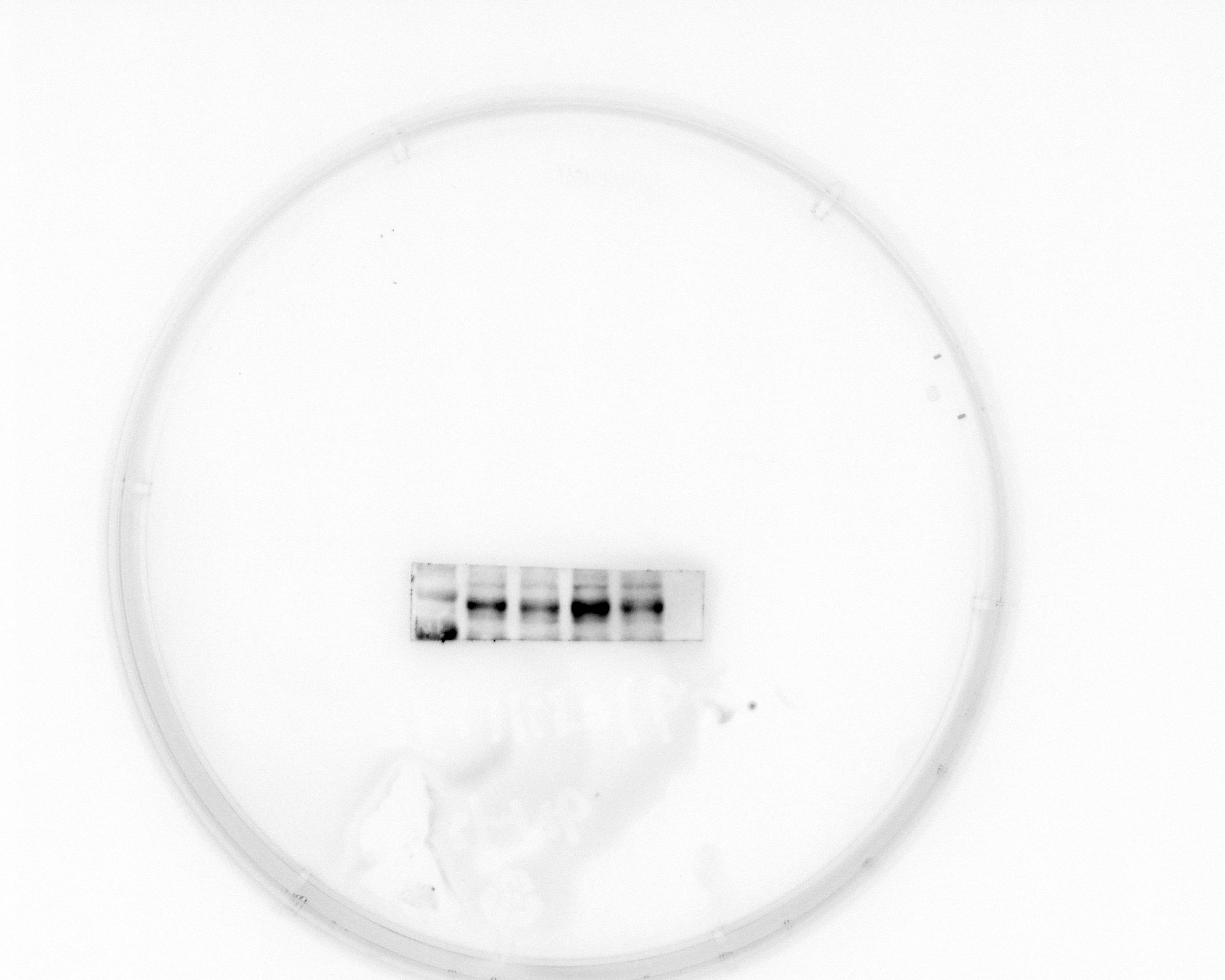

Supplement: Supplementary file 3 [file DataSheet2.zip › Raw data/CF-23.5.31/cell/NEIL3-CELL.jpg]

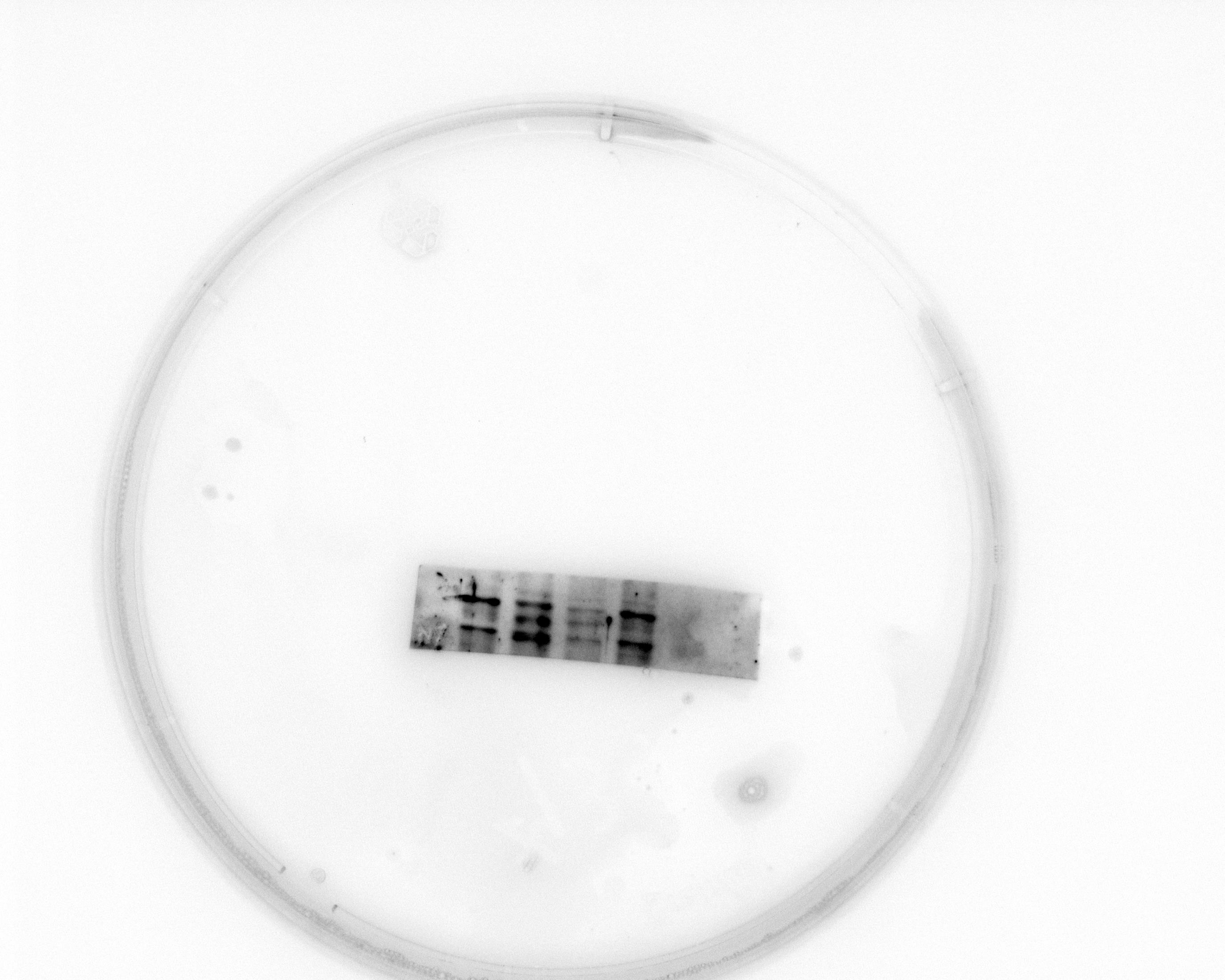

Supplement: Supplementary file 3 [file DataSheet2.zip › Raw data/CF-23.5.31/cell/NT5DC2-HCC.jpg]

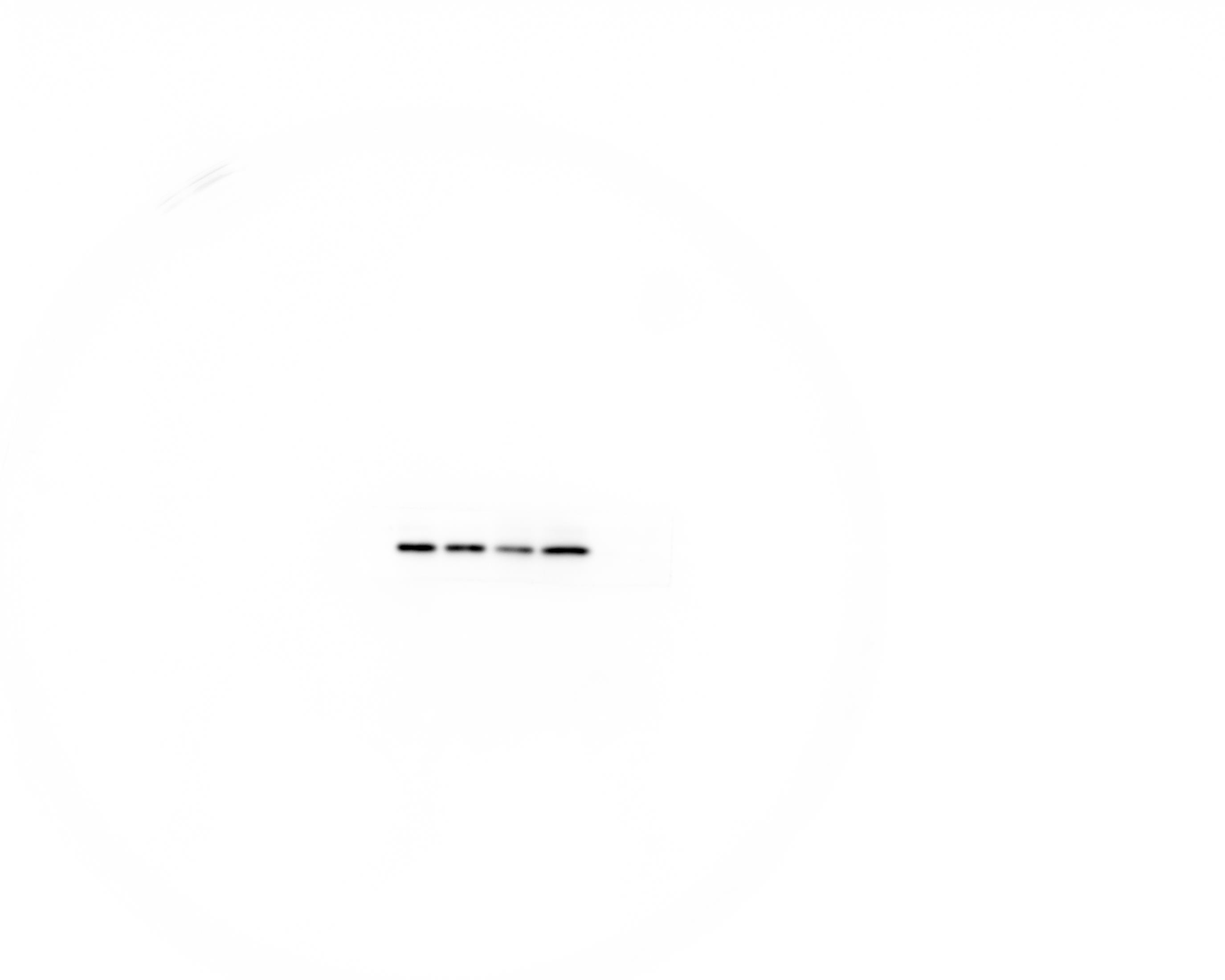

Supplement: Supplementary file 3 [file DataSheet2.zip › Raw data/CF-23.5.31/cell/gclm-hcc.jpg]

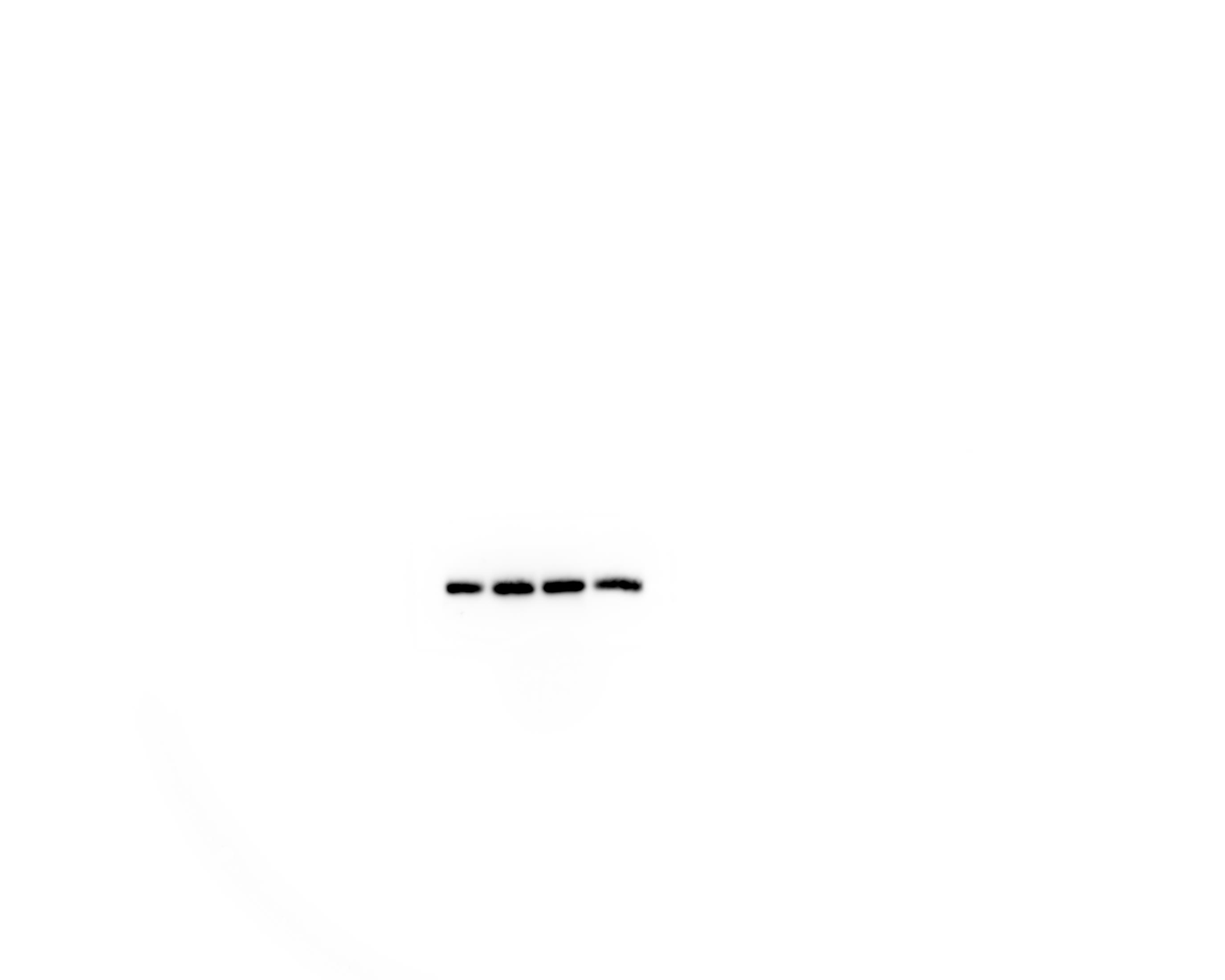

Supplement: Supplementary file 3 [file DataSheet2.zip › Raw data/CF-23.5.31/cell/h3-hcc.jpg]

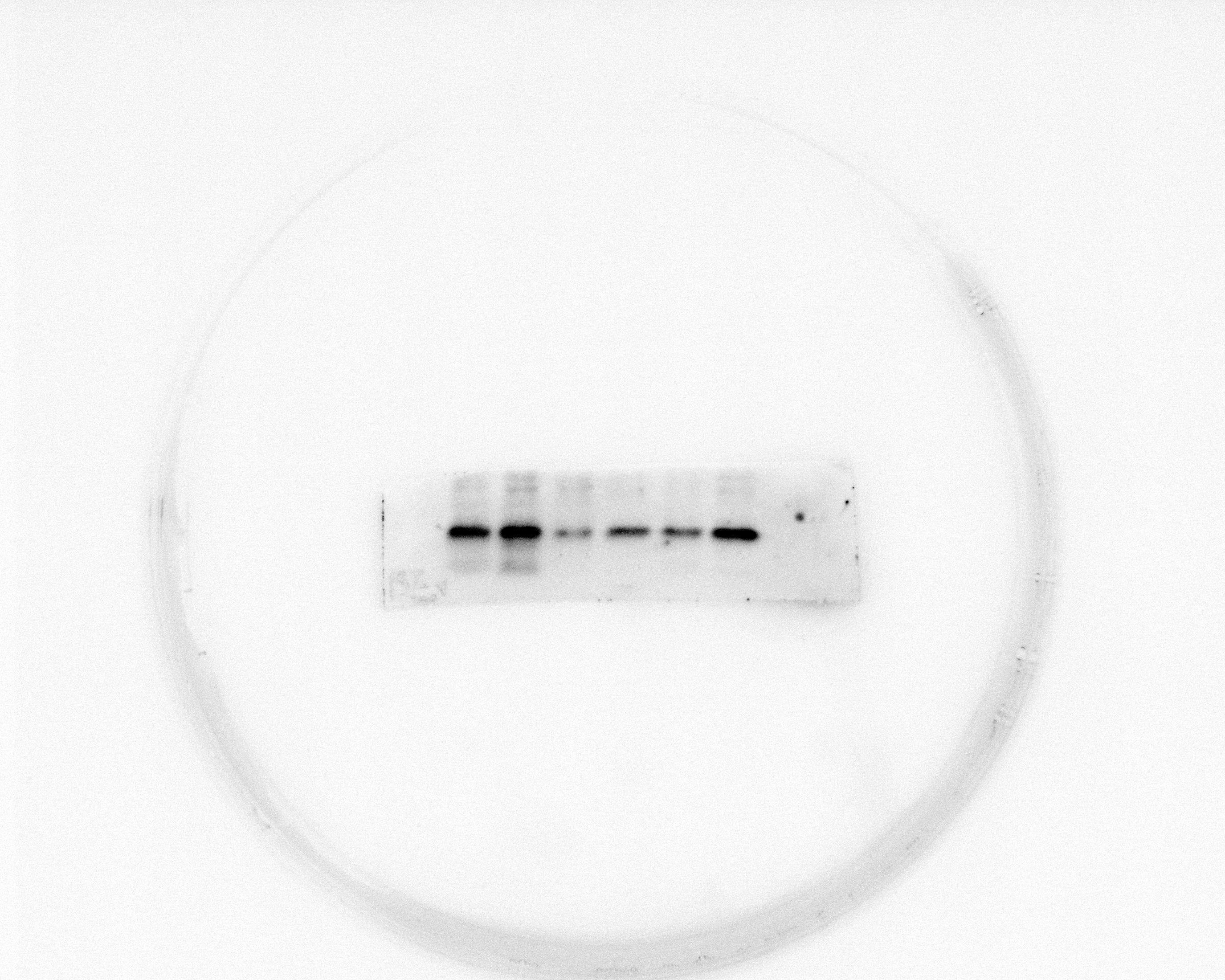

Supplement: Supplementary file 3 [file DataSheet2.zip › Raw data/CF-23.5.31/samples/BEX1-HCC.jpg]

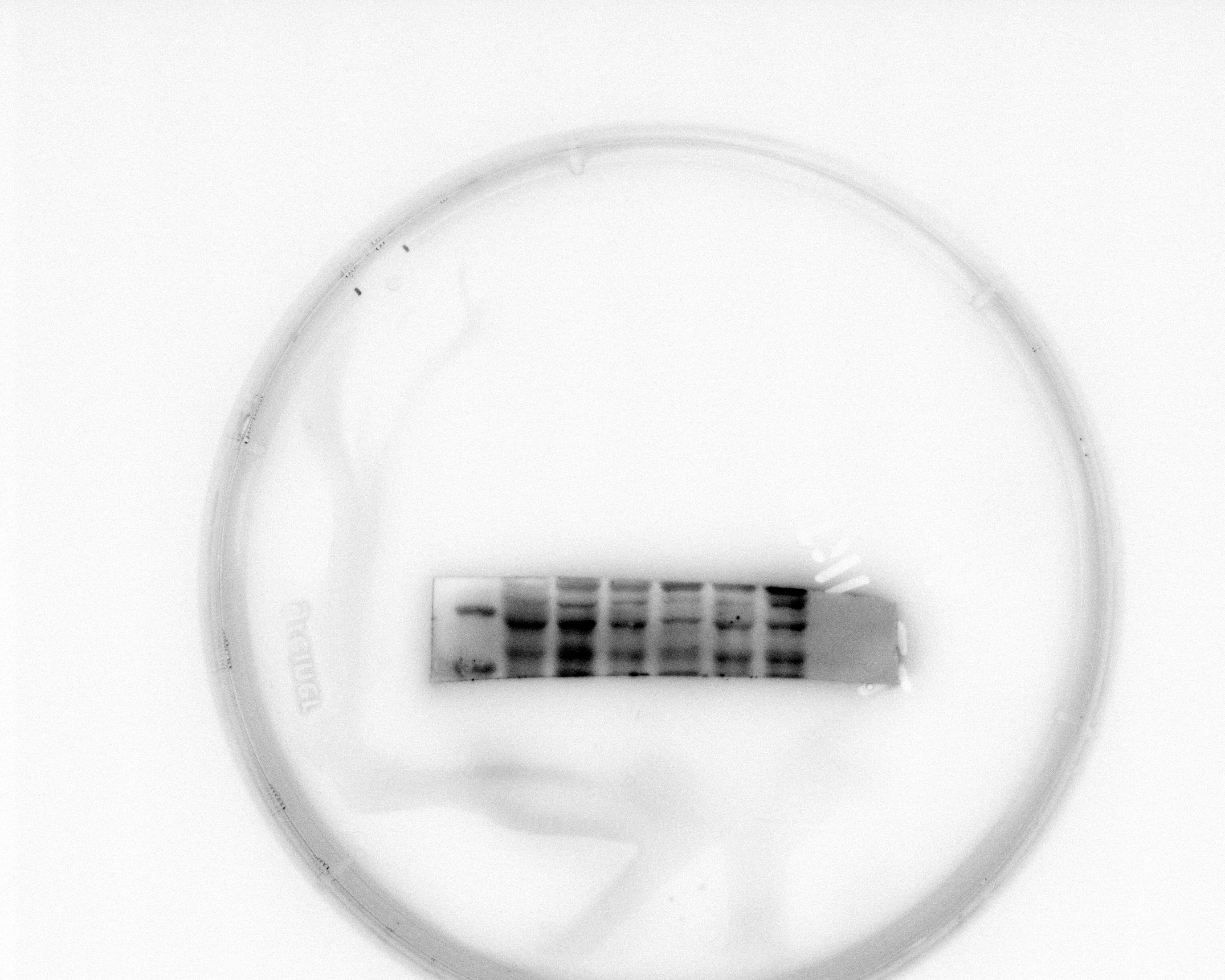

Supplement: Supplementary file 3 [file DataSheet2.zip › Raw data/CF-23.5.31/samples/G6PC-HCC.jpg]

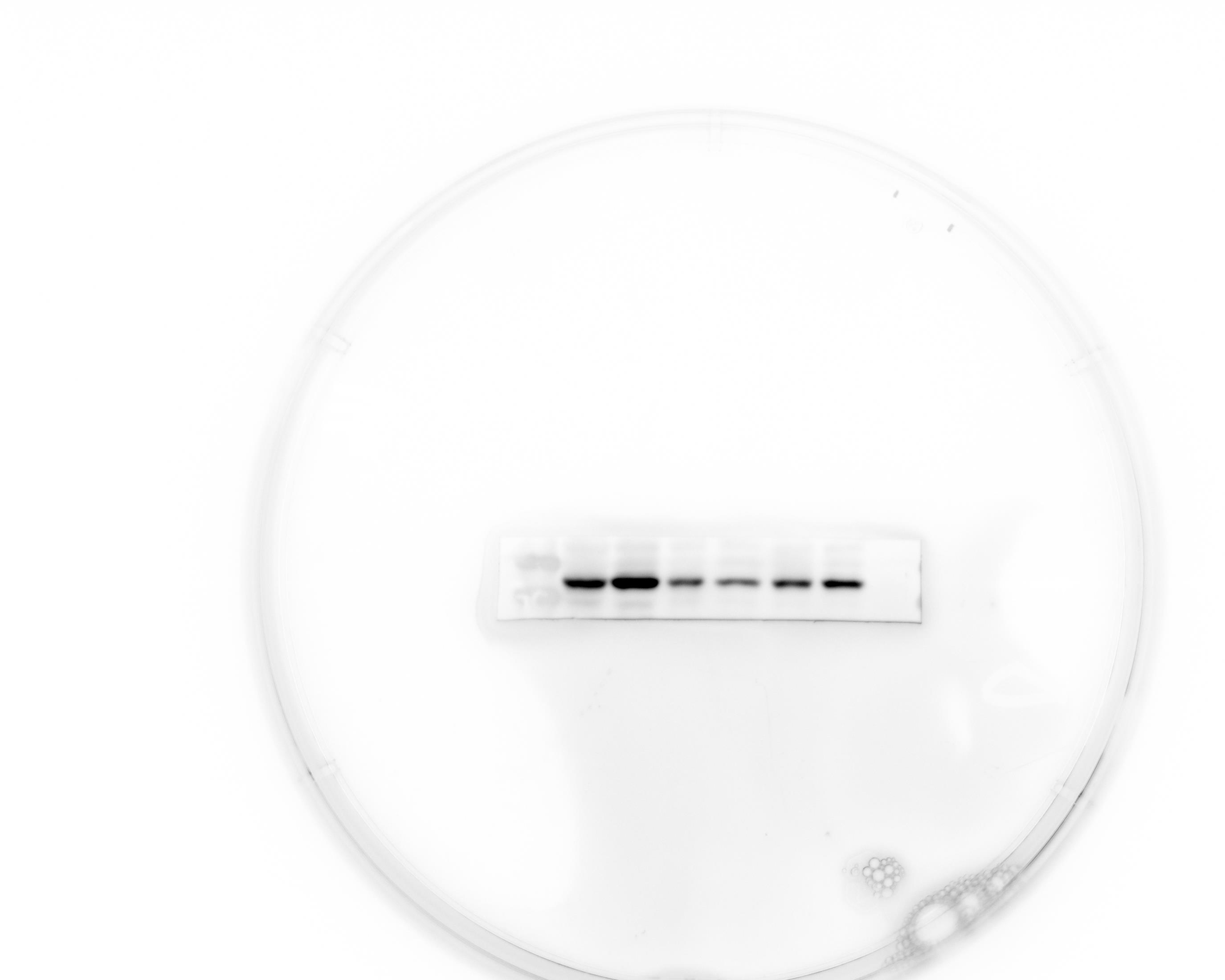

Supplement: Supplementary file 3 [file DataSheet2.zip › Raw data/CF-23.5.31/samples/GCLM-HCC.jpg]

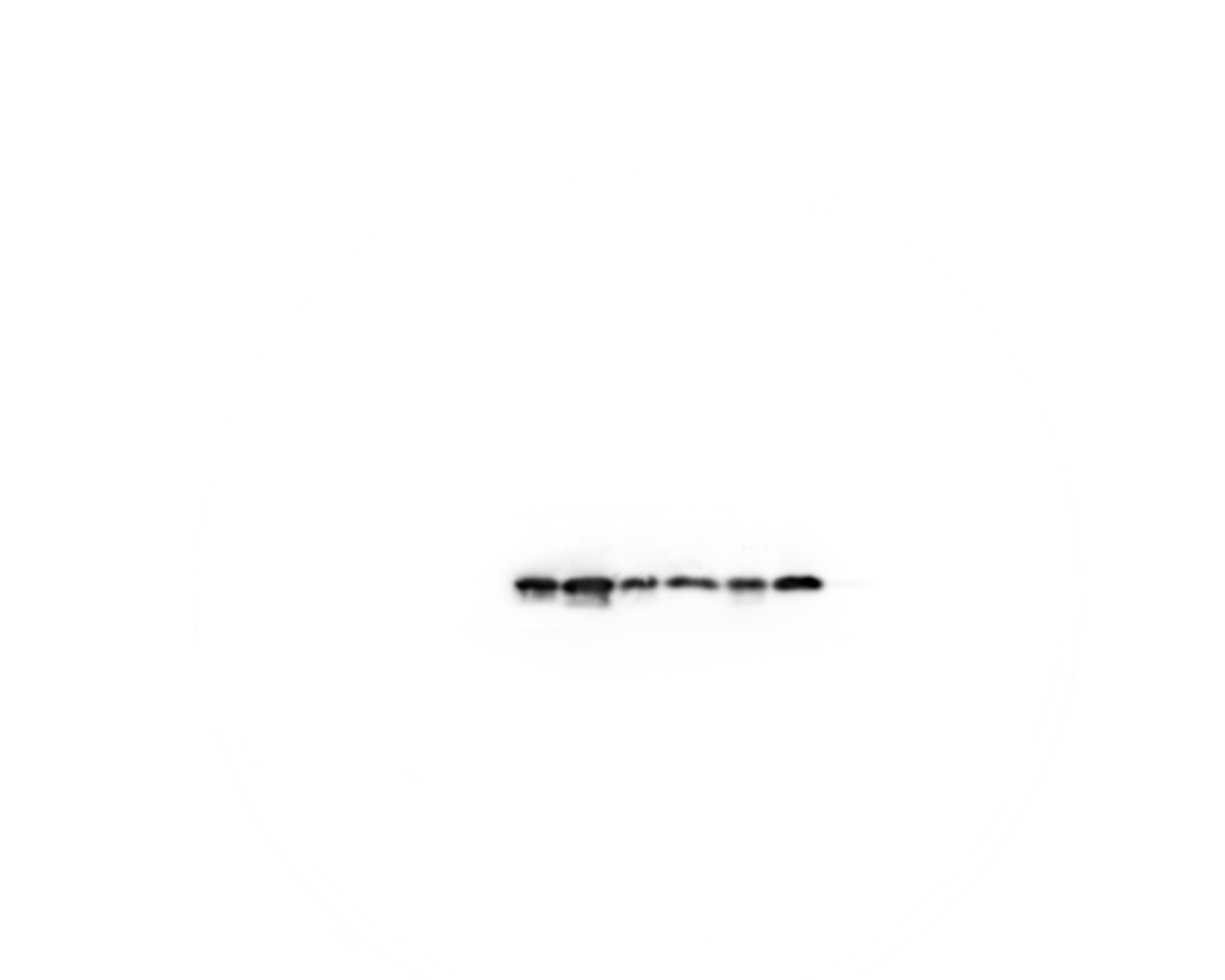

Supplement: Supplementary file 3 [file DataSheet2.zip › Raw data/CF-23.5.31/samples/H3-HCC.jpg]

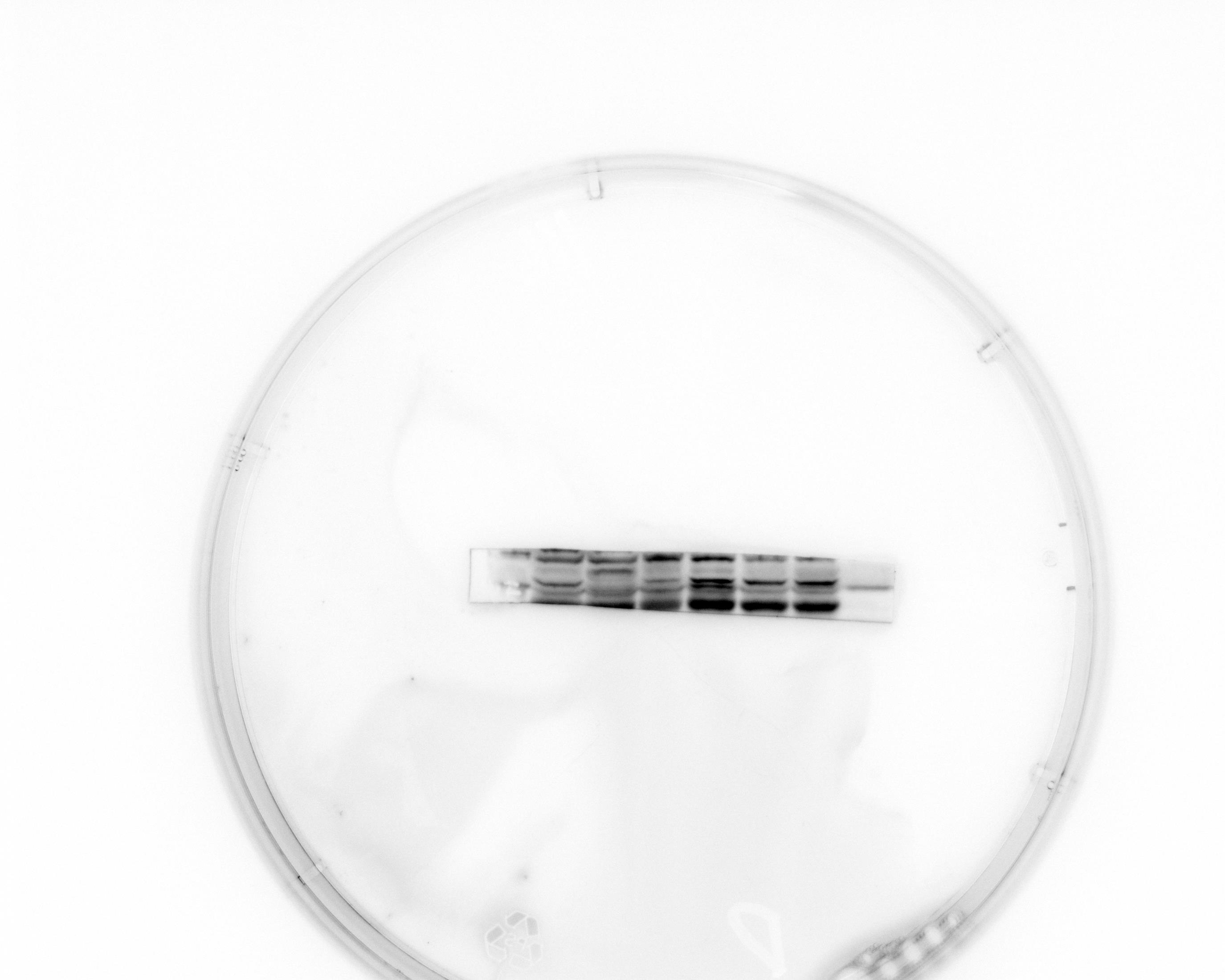

Supplement: Supplementary file 3 [file DataSheet2.zip › Raw data/CF-23.5.31/samples/NEIL3-HCC.jpg]

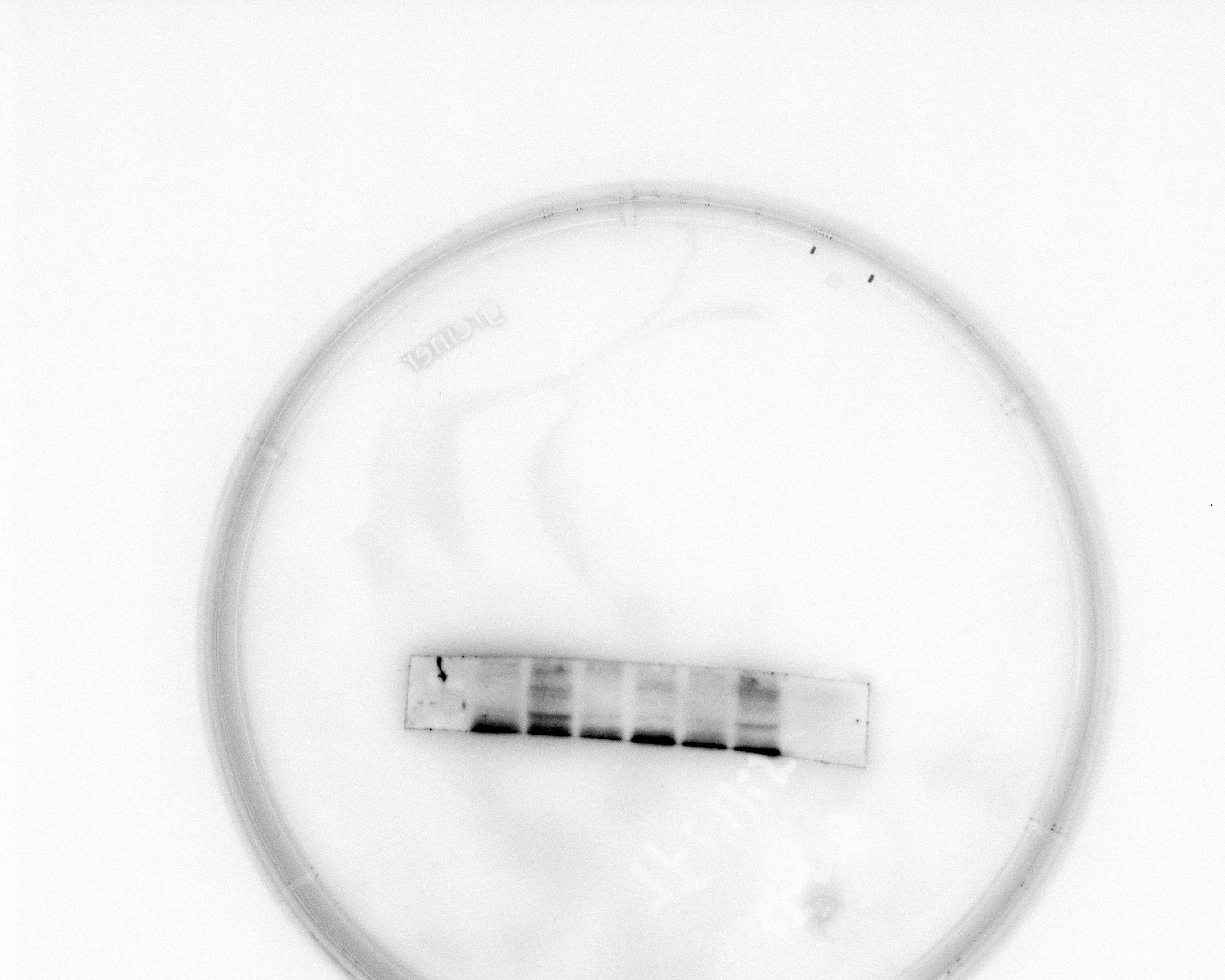

Supplement: Supplementary file 3 [file DataSheet2.zip › Raw data/CF-23.5.31/samples/NT5DC2-hcc.jpg]

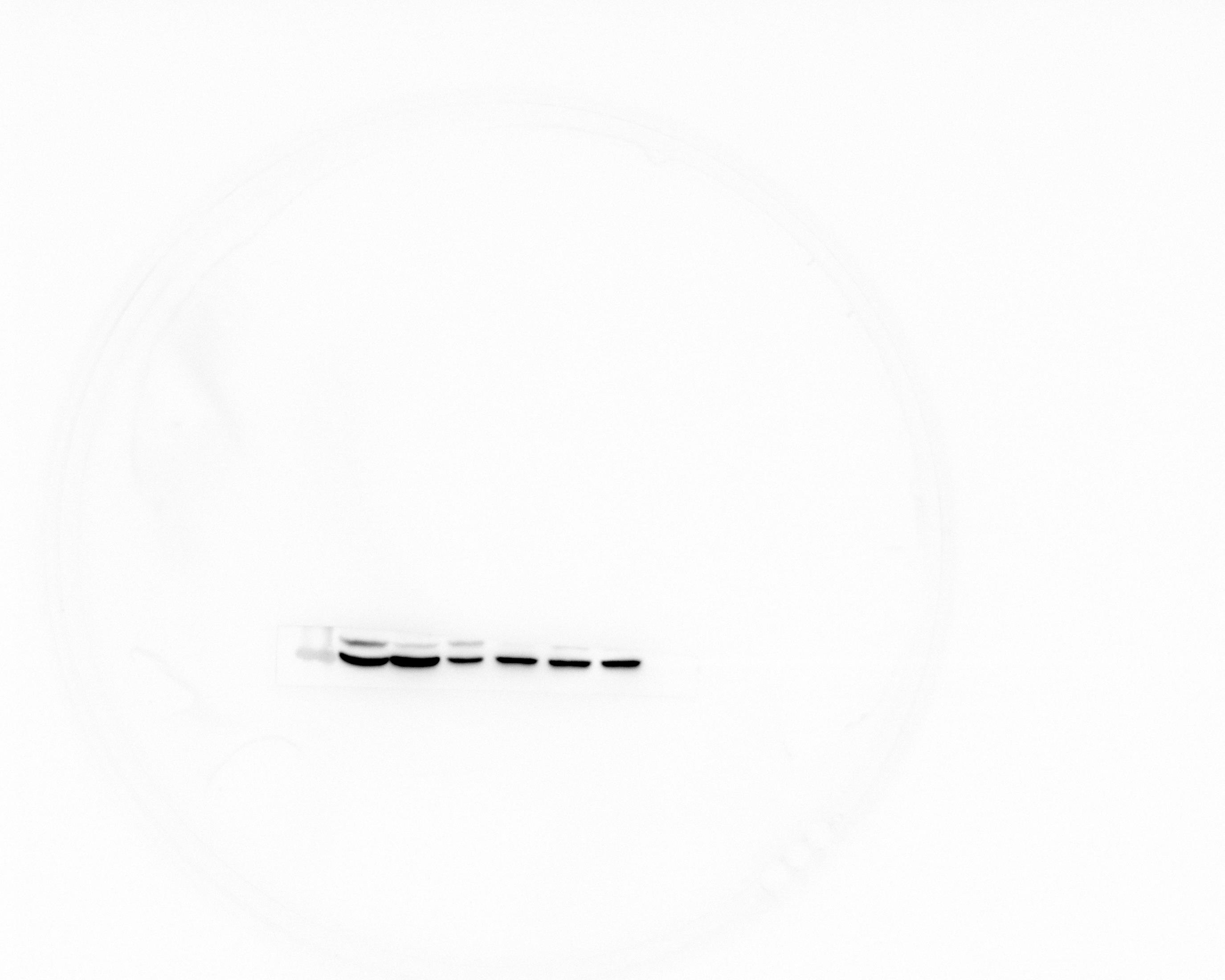

Supplement: Supplementary file 3 [file DataSheet2.zip › Raw data/CF-23.5.31/samples/actin-hcc.jpg]
