# Supplementary material for: Single-cell and genetic multi-omics analysis combined with experiments confirmed the signature and potential targets of cuproptosis in hepatocellular carcinoma
Source: Front Cell Dev Biol. 2023 Sep 8;11:1240390. doi: 10.3389/fcell.2023.1240390 (PMC10516581; doi:10.3389/fcell.2023.1240390)
Supplement: Supplementary file 4 [file DataSheet1.docx]

**Single-cell and genetic multi-omics analysis combined with experiments confirmed the signature and potential targets of cuproptosis in hepatocellular carcinoma**

**Feng Cao^1†^, Yong Qi^2†^, Wenyong Wu^2^, Xutong Li^4*^, Chuang Yang^5^**

Supplementary material


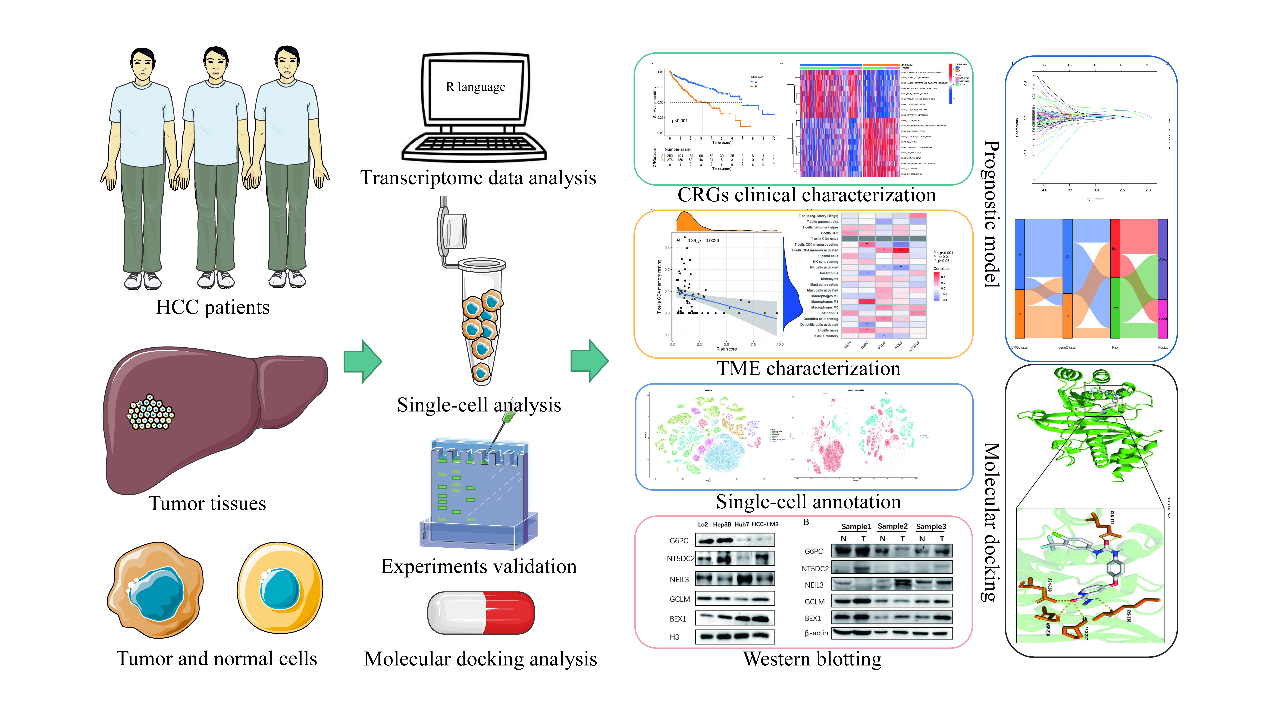


**Figure S1** The entire analytical process of the study.


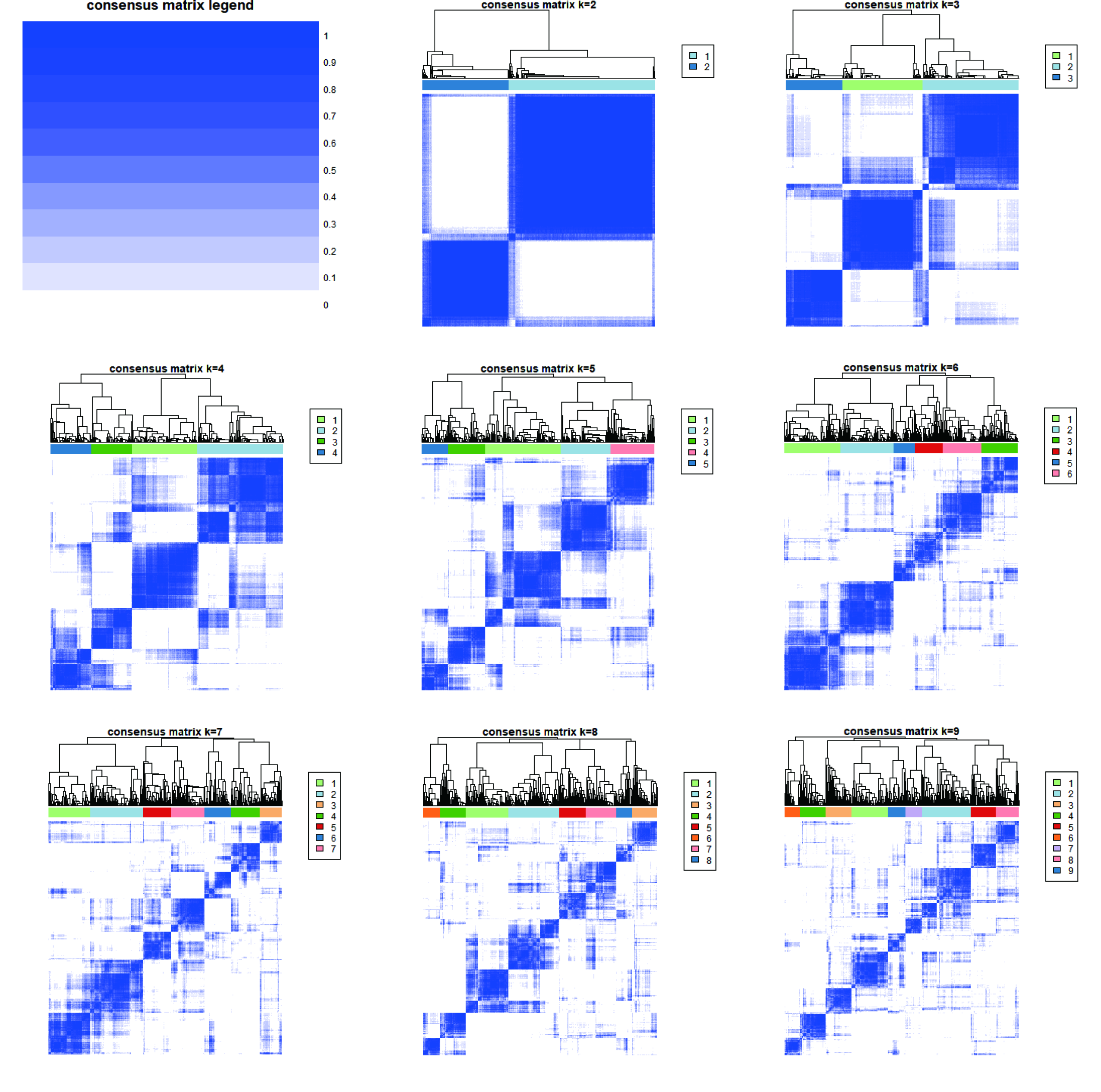


**Figure S2** Unsupervised clustering of curoptosis-related genes and consensus matrix heatmaps for k = 3-9.


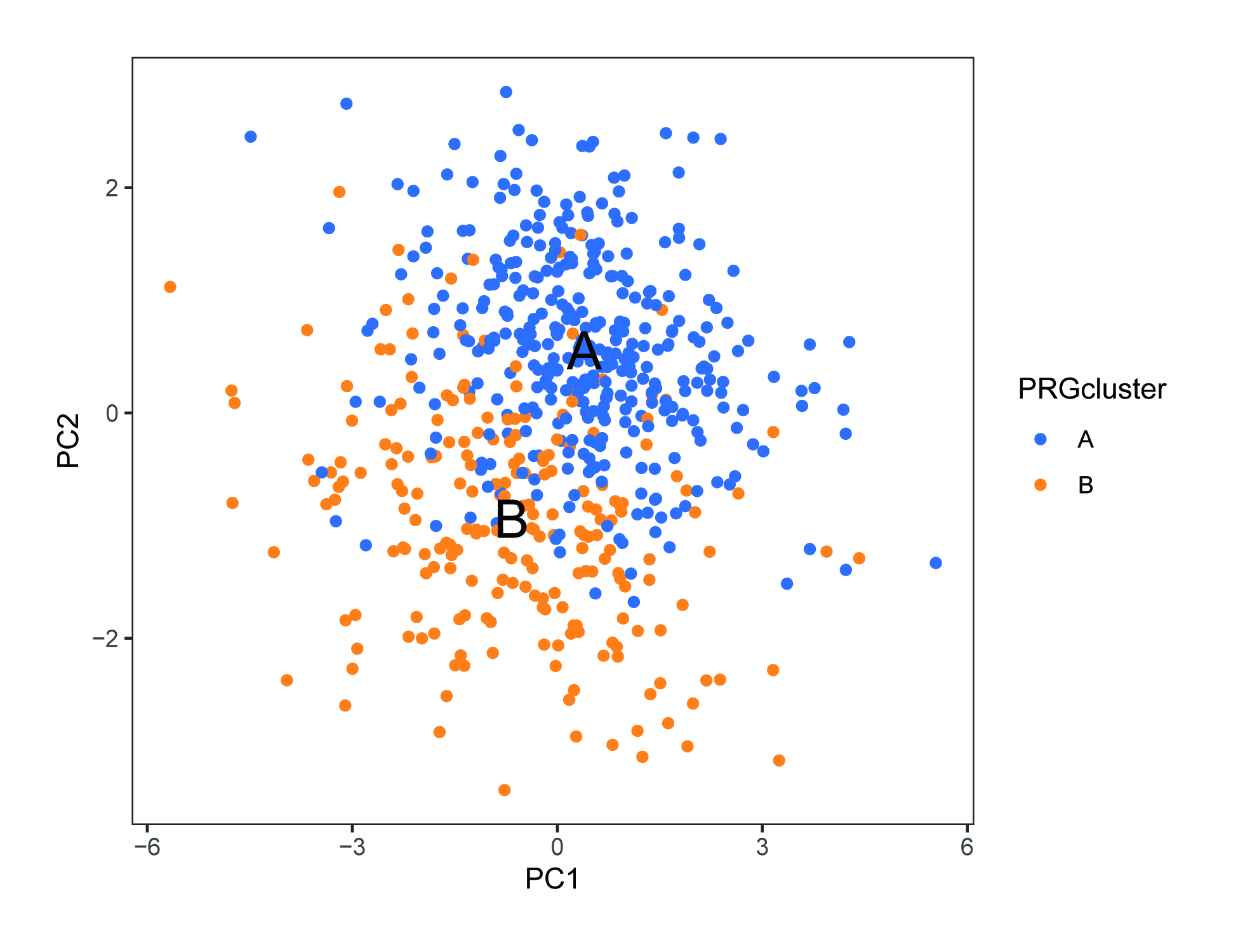


**Figure S3** PCA divided GC samples into two subtypes.


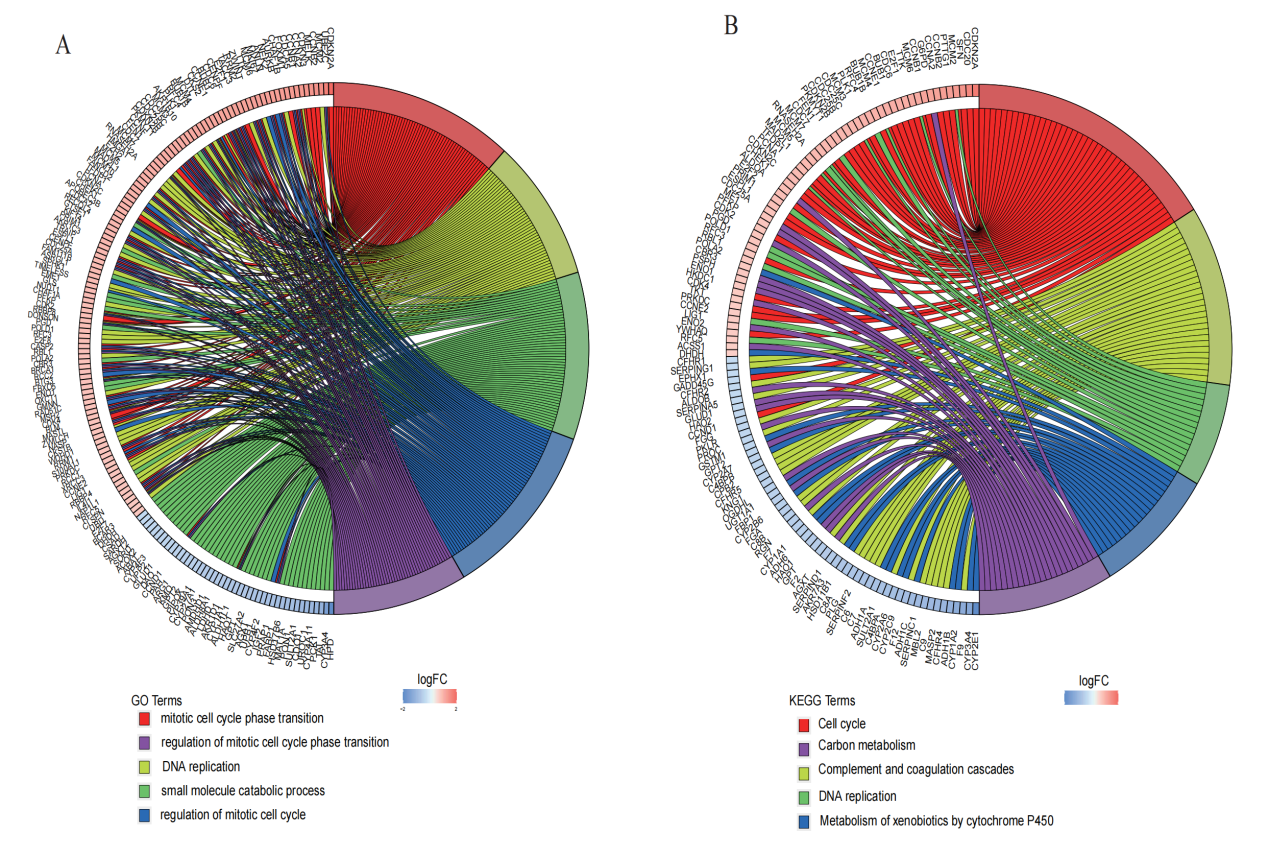


**Figure S4** GO and KEGG enrichment analyses of DEGs.


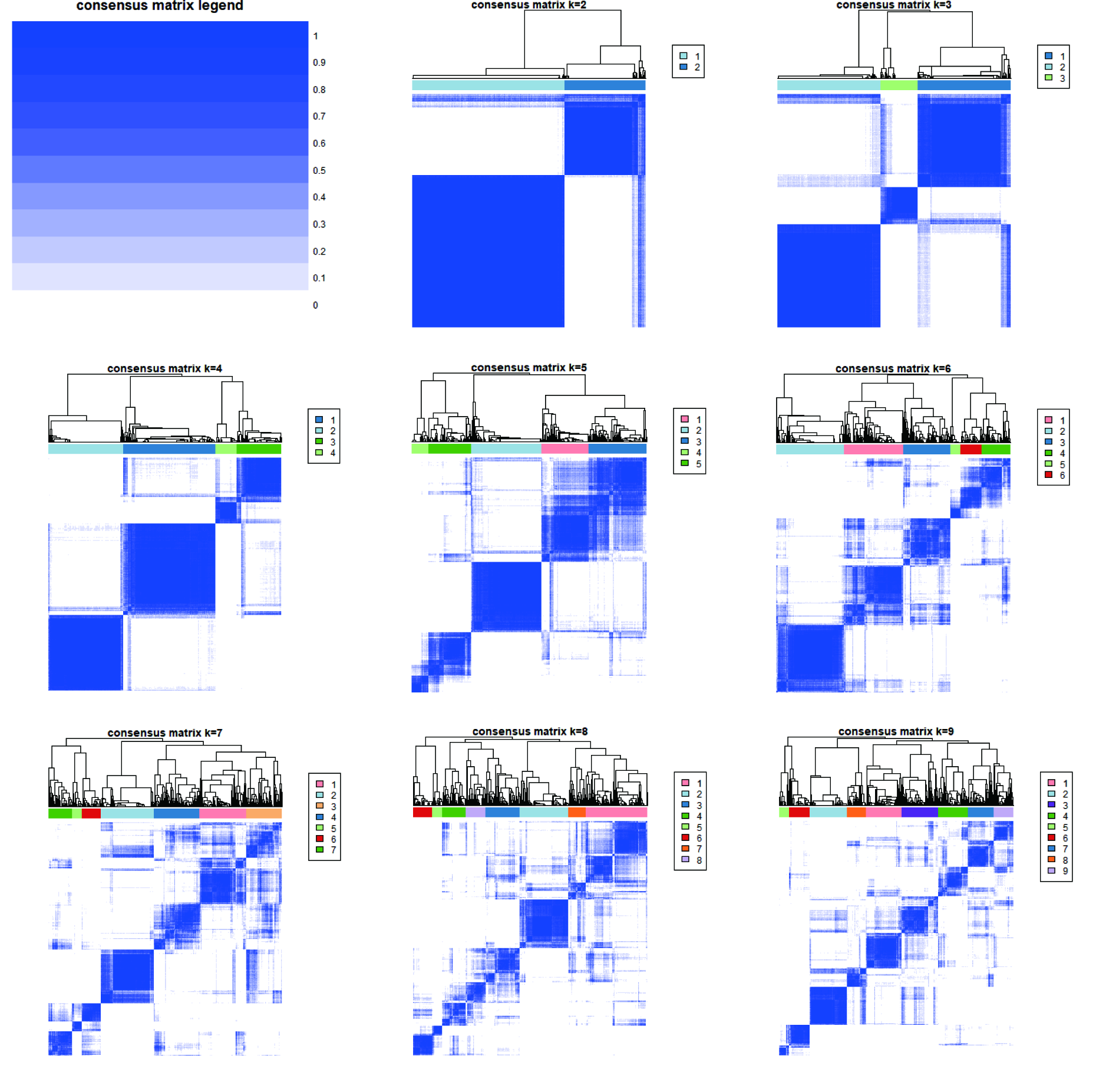


**Figure S5** Unsupervised clustering of CRG-related genes and consensus matrix heatmaps for k = 3-9.


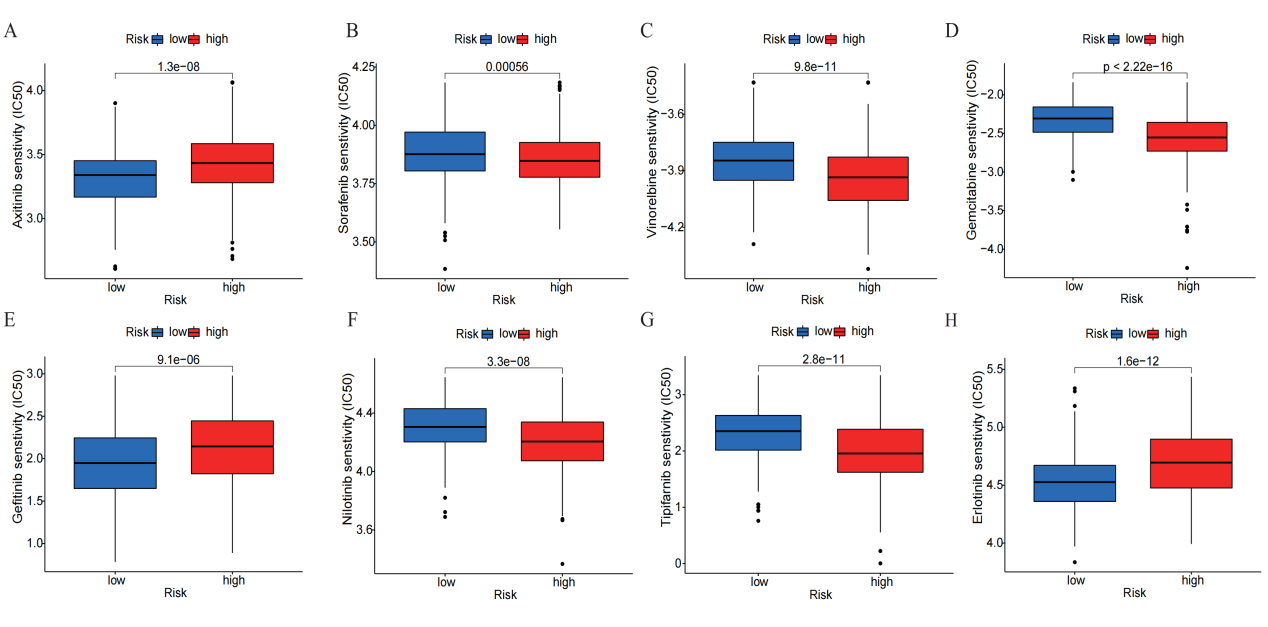


**Figure S6** The differences of chemotherapeutic sensitivity between the high- and low-risk group.


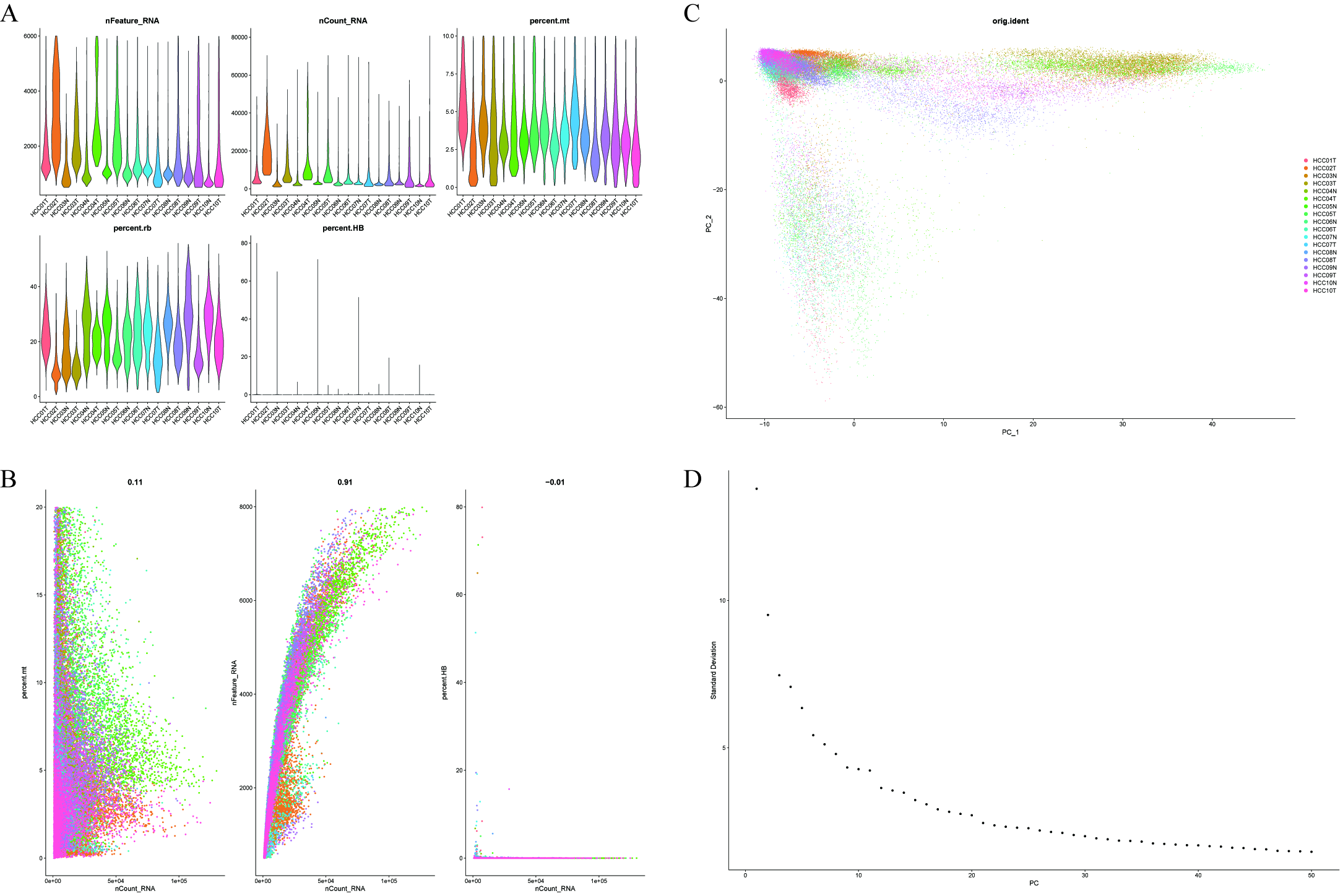


**Figure S7** Preprocessing of the single-cell sequencing data. (A-B) Genes filtering and the correlation between UMIs and mitochondrial ratio, the total number of genes, and the hemoglobin ratio. (C-D) PCA clustering of the genes expression matrix, shown in PC diagram and elbow plots.
